# Comparative study of the effects of different carbon substrates on acidification-driven mineral weathering by *Pseudomonas*

**DOI:** 10.1128/aem.01626-26

**Published:** 2026-09-22

**Authors:** Ségolène Bouche, David Billet, Emmanuelle Morin, Marie-Pierre Turpault, Stéphane Uroz

**Affiliations:** 1 INRAE, UMR1136 Interactions Arbres-Microorganismes, Université de Lorraine 137665, Nancy, France; 2 INRAE, UR1138 Biogéochimie des Écosystèmes Forestiers 27057 , Champenoux, France; 3 CNRS, LIEC, Université de Lorraine 137665, Nancy, France; Colorado School of Mines, Golden, Colorado, USA

**Keywords:** *Pseudomonas*, mineral weathering, acidification, carbon sources, rhizosphere

## Abstract

While carbon sources released by plant roots and fungi are known to favor the enrichment of mineral weathering (MWe) bacteria, their impact on MWe activity remains poorly understood. The relationships among bacterial MWe ability, metabolism, and carbon source utilization, therefore, remain to be elucidated. In this context, a collection of plant growth-promoting *Pseudomonas* strains, including the newly described strain *Pseudomonas spelaei* PML3(3), was screened for its ability to weather minerals [i.e., Ca_3_(PO_4_)_2_ and biotite]. The impact of 10 carbon sources was assessed, together with the underlying genetic basis of these strains, by combining *in vitro* bioassays and geochemical and *in silico* analyses. The strains were ranked according to their MWe abilities in the different assays. All the strains were able to weather at least one mineral type, and their effectiveness depended on the substrate metabolized, with the strain PML3(3) generally being the most effective. Comparative genomic analyses linked the ability of the different strains to acidify the solution and produce organic acids, such as gluconate, to specific genomic organizations. Further analyses revealed that acidification-driven MWe was delayed when mannitol, rather than glucose, was used as the sole carbon source, suggesting that, depending on the substrate, either a single pathway, such as the direct oxidative glucose pathway, or multiple metabolic pathways may operate simultaneously to confer mineral weathering ability to bacteria. Our new findings demonstrate that MWe by bacteria strongly relies on carbon metabolism and represents a more dynamic and complex process than previously expected.

**IMPORTANCE:**

In this work, we investigated the acidification-driven mineral weathering (MWe) mechanism and its genetic basis in a collection of *Pseudomonas* strains, including the model strain *Pseudomonas spelaei* PML3(3). Comparative genomic analyses revealed two major genomic organizations associated with the direct oxidative (DO) pathway of glucose that are commonly found within the genus *Pseudomonas*: (i) a system comprising only a pyrroloquinoline quinone (PQQ)-dependent glucose dehydrogenase (GDH) and (ii) a system comprising a PQQ-dependent GDH and a FAD-dependent gluconate dehydrogenase (GADH). These results indicate that the enzymes involved in the DO pathway of glucose are broadly conserved across the genus. Our results revealed intragenus variability in the MWe abilities of *Pseudomonas* strains isolated from diverse environments. *In vitro* assays [i.e., Ca_3_(PO_4_)_2_ and biotite dissolution assays and acidification assays] revealed phenotypic differences that depended on the carbon source, the bacterial strain, and the type of mineral. Four phenotypic patterns were observed: (i) high MWe activity associated with strong acidification and limited growth; (ii) in the tricalcium phosphate (TCP) assay only, moderate MWe activity beneath the colony associated with good bacterial growth; (iii) no MWe activity associated with the absence of acidification and strong growth; or (iv) complete absence of growth and MWe activity. These findings support a close relationship between carbon source utilization, bacterial metabolism, and MWe effectiveness. This work advances our understanding of bacterial mineral weathering, a dynamic process that plays a key role in long-term soil fertility and plant growth and whose effectiveness is influenced by multiple factors, including the nature of the mineral substrate.

## INTRODUCTION

Over time, plants have continually had to adapt to changing environments with strong differences in terms of climate, soil, and microbiota. This has driven extensive plant diversification. Beyond their genetic specificities, it is now recognized that a long coevolution has occurred between plants and soil microorganisms (1). Notably, some chemical properties attributed to plants or fungi have been shown to result from molecules (e.g., alkaloids) produced by their associated microbiota (2–5). A broad range of microorganisms have been reported to interact with plants, colonizing their tissues and, with some of them, establishing symbiotic associations (e.g., fungi, bacteria, and oomycetes) (4). The plant microbiota is considered to play key roles in nutrition, protection against pathogens, resistance to environmental stresses (e.g., drought, extreme temperatures, and salinity) (6, 7), and even the adaptation and evolution of their host (8).

The rhizosphere remains the most extensively studied interface between plants and their environment, particularly with respect to plant–microorganism interactions. Within this plant–soil interface, a selective enrichment of specific taxa and functions occurs (9–11). However, this enrichment depends on the soil conditions, the plant species, and the season (12–14). These changes are attributed to the ability of plants to modulate their root exudates according to their local environment, particularly according to the stresses they experience and their nutritional requirements (15, 16). In this sense, plants growing in nutrient-depleted environments have been shown to produce higher levels and different types of compounds than when they grow in nutrient-rich environments (16, 17).

Studies have shown that heterotrophic rhizosphere bacteria are more effective in metabolizing carbon substrates than those from the surrounding bulk soil (12, 16–19). This enhanced ability is particularly associated with the utilization of plant- and fungal-derived compounds (e.g., fructose, glucose, sucrose, xylose, and succinate) (20, 21) or compounds released by fungi (e.g., mannitol and trehalose for mycorrhizal roots) (22). In contrast, more complex substrates are particularly degraded by bacteria from the surrounding bulk soil (23). The carbon sources released by plant roots and fungi or found in the byproducts of organic matter decomposition are consequently considered the main drivers explaining the distribution and activities of rhizosphere bacterial communities. The effectiveness in weathering minerals has also been shown to characterize rhizosphere bacteria. MWe bacteria have been shown to be particularly effective and more frequent in nutrient-poor environments (e.g., forests, mangroves, and deserts) and in the rhizosphere than in nutrient-rich environments (12, 13, 24–26). Beyond this indirect demonstration of the impact of root exudates on MWe effectiveness, the direct impact of the carbon sources consumed has been shown for several bacterial strains (18, 27, 28). However, the range of carbon substrates tested has usually been limited (27, 29–32). These differences raise questions about the mechanisms underlying bacterial MWe and the potential link between metabolic properties, carbon substrates consumed, and MWe effectiveness.

The ability to weather minerals has been reported across a broad range of bacterial taxa (e.g., *Acinetobacter*, *Bacillus*, *Burkholderia-Caballeronia-Paraburkholderia*, *Collimonas*, *Paenibacillus*, *Pantoea*, and *Pseudomonas*), whose presence and abundance vary strongly depending on the land use, soil conditions, and plant species. Representatives of the genus *Pseudomonas* have been frequently reported in agricultural soils (with usually neutral pH), whereas metagenomic analyses have highlighted their low occurrence in acidic soils (33–35). To date, a limited number of culturable representatives have been isolated from the (mycor)rhizosphere or mineralosphere in acidic soils (12, 18, 36). Consequently, our knowledge of the functional and physiological properties of *Pseudomonas* isolates from acidic soils remains limited. Their potential specificities compared with *Pseudomonas* isolates from agricultural soils and the conservation of this function within the genus remain key questions. Historically, the chemical reactions involved (i.e., the conversion of glucose into protons and gluconate [GA]), their cellular localization (i.e., the periplasm), and the cofactors and enzymes involved (i.e., PQQ and PQQ-dependent glucose dehydrogenase [GDH-PQQ]) have been characterized mainly in *Pseudomonas*, *Pantoea*, and *Acinetobacter* strains isolated from agricultural soils (37). On the basis of these findings, the DO pathway of glucose (Step 1: conversion of glucose to GA and H^+^) plays a key role in the acidification-driven MWe process and can continue with the conversion of GA to 2-ketogluconate (2KGA) and H^+^ (Step 2). This second reaction is performed by a gluconate dehydrogenase (GADH), a flavin adenine dinucleotide (FAD)-dependent enzyme belonging to the glucose–methanol–choline (GMC) oxidoreductase family (38, 39). In *Pseudomonas*, two scenarios have been observed regarding glucose oxidation: (i) an oxidation pathway restricted to Step 1 or (ii) an oxidation pathway involving both Step 1 and Step 2 (40, 41).

In this context, we aimed to test the central hypotheses that: (i) the effectiveness in weathering minerals through the acidification-driven MWe mechanism depends on the carbon substrate metabolized, (ii) the kinetics of mineral dissolution varies according to the carbon substrate metabolized, and (iii) the genomic potential (presence of *gdh-PQQ*/*gadh-FAD*) does not strictly predict MWe ability across diverse *Pseudomonas* isolates from different soil origins. In our study, we used *Pseudomonas spelaei* PML3(3) as a model strain. This strain, which was isolated from acidic forest soil, was selected to compare its MWe ability and genomic organization with those of reference *Pseudomonas* strains that originated primarily from agricultural environments. Using a collection of culturable strains previously characterized for their effectiveness at solubilizing inorganic phosphorus using glucose and/or at protecting plants against pathogens and/or at promoting plant growth (PGP), we compared the effectiveness in weathering, the genetic basis, and the impact of the carbon source consumed. To achieve this goal, we combined *in vitro* assays and (geo)chemical analyses to determine the effectiveness in weathering and identify the main organic acids produced by a collection of 10 *Pseudomonas* strains encompassing the strain PML3(3). *In silico* analyses were also performed to assess the presence of putative genes encoding the PQQ cofactor, GDH-PQQ, and GADH-FAD in the genomes of a broader range of *Pseudomonas* strains.

## MATERIALS AND METHODS

### Bacterial strains and culture media

A total of 10 model strains of *Pseudomonas* were used in this study (see the list in Table S1). All the strains were isolated from plant environments [i.e., the (mycor)rhizosphere or phyllosphere] and have previously been characterized for different plant growth-promoting traits (42–47). Most strains originated from laboratory collections or were kindly provided by collaborators. Strains were selected according to three criteria: (i) demonstrated plant and/or fungal growth-promoting or biocontrol effects, (ii) available genome sequences, and (iii) the ability to solubilize phosphorus. For some of these strains, the genetic basis supporting their ability to solubilize inorganic P through an acidification-driven MWe mechanism was demonstrated. The model strain used in this study was *Pseudomonas spelaei* PML3(3) (48). This strain has been isolated from the *oak/Scleroderma citrinum* ectomycorrhizosphere in the acidic forest soil of the Breuil–Chenue experimental site (France) (23) and represents one of the few *Pseudomonas* strains isolated from this site. All the bacterial strains were grown at 25°C on a modified version of Luria–Bertani (LBm) medium containing a reduced salt concentration (g·L^−1^: 10, tryptone; 5, yeast extract; 1, NaCl) and supplemented with 15 g·L^−1^ of agar. The bacterial strains were cryopreserved at −80°C in 40% glycerol.

### Mineral description

Biotite was selected for this study because it is present in the granite of the Breuil–Chenue site (49). It is a 2:1 phyllosilicate frequently present in acidic soils, where it undergoes relatively rapid weathering and releases nutrients involved in tree nutrition, including K, Mg, and Fe. The biotite used is a pure homogeneous mineral from Bancroft (Canada). Its chemical composition was as follows (g·kg^−1^): 410.1, SiO_2_; 109, Al_2_O_3_; 22.1, Fe_2_O_3_; 100.5, FeO; 2.7, MnO; 189, MgO; 4.1, Na_2_O; 94.6, K_2_O; 22.8, TiO_2_; 44.2, F; and 0.8, Zn. Its structural formula is (Si_3_Al_1_) (Fe^3+^_0.12_ Fe^2+^_0.61_ Mg_2.06_ Mn_0.02_ Ti_0.13_) K_0.88_ Na_0.06_ O_10_ (OH_0.98_ F_1.02_). To prepare the biotite particles used in this study, the biotite crystals were first fragmented and inspected with a stereomicroscope to exclude impurities (i.e., calcite, quartz, and iron oxides). Afterward, they were ground and subjected to ultrasonication (2 min, three times at 100 V) to remove the fine particles that electrostatically adhered to the particles. After washing three times with distilled water, the material was sieved to obtain a calibrated particle size range of 200 to 500 μm.

### Preparation of bacterial inoculum

For all the functional assays described below, a bacterial inoculum of each strain was prepared using the same procedure. After 3 days of incubation in 10 mL of liquid LBm medium at 25°C, the cultures were centrifuged at 7,000 × *g* for 15 min, and the pellet was subsequently washed three times with the same volume of sterile Milli-Q water. The pellets were ultimately resuspended in sterile Milli-Q water, and the optical density at 595 nm (OD_595_nm) was adjusted to 0.90 ± 0.04 [corresponding to ~6 × 10^8^ cells mL^−1^ for strain PML3(3)]. The relationship between the OD_595_nm measure and the number of cells was determined by dilution plating on LBm agar.

### Phenotypic characterization of the bacterial strains

To determine the ability of the different strains to consume different carbon substrates, their metabolic profiles were determined using a BIOLOG GEN Ш MicroPlate (Biolog, Hayward, CA, USA). This assay is the only one for which the inoculum was adjusted to an OD_595_nm of 0.60 ± 0.04 [corresponding to ~3 × 10^8^ cells mL^−1^ for the strain PML3(3)] to avoid absorbance saturation after incubation. A volume of 100 μL of this inoculum was inoculated into each well of the BIOLOG GEN Ш MicroPlate. After 2 days of incubation at 25°C, the OD_595_nm was measured, and the data were analyzed according to the manufacturer’s instructions.

### Mineral weathering assays

Given that our study focused on the acidification-driven MWe mechanism developed by the different *Pseudomonas* strains, culture conditions without strong buffering capacity were used. Indeed, studies have clearly demonstrated that the presence of buffer strongly limits the development and impact of acidification by effective MWe bacteria (50). Different culture conditions were considered to assess their ability to (i) acidify the solution, (ii) solubilize inorganic phosphorus, and (iii) weather biotite. Under each condition, the effect of the carbon source metabolized was additionally considered. A total of 10 substrates (i.e.*,* glucose, galactose, fructose, xylose, trehalose, sucrose, mannitol, sorbitol, gluconate, and succinate) were considered because (i) they are released by roots and fungi, (ii) they are constituents of fungal and plant cells, or (iii) they represent degradation byproducts of glucose or plant constituents.

#### Acidification assay

An iron-depleted version of the Bushnell–Haas medium (BHm) (18) was used to determine the acidification capacity of the strains. Its composition (in g·L^−1^) is 0.02, KCl; 0.15, MgSO_4_·7H_2_O; 0.080, NaH_2_PO_4_·2H_2_O; 0.090, Na_2_HPO_4_·2H_2_O; 0.065, (NH_4_)_2_SO_4_; 0.1, KNO_3_; and 0.020, CaCl_2_·2H_2_O (pH adjusted to 6.5). The medium was supplemented with a carbon substrate at 2 g·L^−1^. Briefly, 20 μL of bacterial inoculum was added to 180 μL of BHm (with different carbon sources) in a 96-well microplate. After 3 days of incubation at 25°C under 150 rpm orbital agitation, the microplates were centrifuged at 1,000 × *g* for 15 min at 20°C. A volume of 180 μL of the culture supernatant was added to a new microplate containing 20 μL of bromocresol green dye (1 g·L^−1^). The pH was determined by measuring the OD_595_nm and converting the values using a calibration curve (18). The values presented correspond to the mean of three replicates.

#### Inorganic phosphorus solubilization assay

Inorganic phosphorus (P) solubilization was assessed using solid tricalcium phosphate (TCP) medium. The medium composition was as follows (in g·L^−1^): 5, NH_4_Cl; 1, NaCl; 1, MgSO_4_·7H_2_O; 4, Ca_3_(PO_4_)_2_; and 15, agar (the pH was adjusted to 6.5). The medium was supplemented with a carbon substrate at 10 g·L^−1^. Briefly, 5 μL of bacterial inoculum of each strain was spotted onto TCP agar plates in triplicate and incubated for 14 days at 25°C. The diameter of the colonies (ØC) and solubilization halo (ØH) was measured. Bacterial growth on TCP agar plates resulted in the formation of colonies that, depending on the physiological properties of the strain (e.g., motility or exopolysaccharide production) and the conditions, exhibited variable colony morphologies (e.g., convex or spreading). The presence of halos beneath colonies was also determined by washing the TCP plates with Milli-Q water. A volume of 5 mL of sterile Milli-Q water was added to each Petri dish, and the colonies were removed using a cell spreader. This combined approach allowed us to determine whether acidification-driven MWe occurred remotely (i.e., halo formation around colonies) or locally (i.e.*,* halo formation beneath colonies). The carbon substrate concentration used in this assay was higher than that in the other assays (i.e., in BHm) because the formation of solubilization halos on TCP plates requires a longer incubation time.

#### Biotite weathering assay

The mineral weathering potential of each bacterial strain was determined using 96-well microplates prefilled with biotite, as described by Uroz et al. (18). Briefly, 20 μL of bacterial inoculum was added to sterile MultiScreen microplates (MAGVN22, 0.22 mm pore size; Millipore) containing 10 mg of sterile biotite particles and 180 µL of BHm medium supplemented with a carbon substrate at 2 g·L^−1^. After 3 days of incubation at 25°C and under 150 rpm orbital agitation, the MultiScreen microplates were centrifuged at 1,000 × *g* for 15 min. The filtrates were transferred to a new microplate containing 20 μL of bromocresol green dye (1 g·L^−1^) for pH determination in the first 4 wells of the column and 20 μL of Spectroquant (ref. 1.14761.0001, Supelco) for iron quantification in the next 4 wells of the column. The pH and concentration of iron in the solution were determined by measuring the OD_595_nm and converting the data obtained using calibration curves (18). A 3-day incubation period was selected because it corresponds to the optimal period for acidification-driven MWe by the different strains under our experimental conditions. The strains *Paraburkholderia* PN3(3) and *Caballeronia* PML1(12) were used as negative and positive controls, respectively (18). Four replicates were performed for iron quantification and for pH measurements for each strain. The data generated were compared to previously generated abiotic data that mimic the acidolysis and acidocomplexolysis mechanisms (18). Acidolysis refers to the MWe mechanism occurring through the massive release of protons and the production of organic acids. Acidocomplexolysis corresponds to a combination of acidolysis (i.e., proton-mediated dissolution) and complexolysis (i.e.*,* dissolution mediated by complexing agents, such as organic acids and siderophores).

### Growth kinetics and organic acid production

To investigate the relationship between the carbon source consumed, bacterial growth, and effectiveness in weathering of each *Pseudomonas* strain, growth and pH were monitored through kinetic analysis. Three time points (i.e., 24, 48, and 72 h) were considered for a subset of the carbon substrates, yielding contrasting results in terms of acidification (i.e., glucose, mannitol, xylose, and gluconate). Additional time points (i.e., 0, 4, 8, 32, and 56 h) were also considered for mannitol and glucose. For each strain, 5 mL of inoculum was added to a 100 mL Erlenmeyer flask containing 45 mL of liquid BHm supplemented with glucose or mannitol (2 g·L^−1^). An abiotic condition consisting of 5 mL of Milli-Q water mixed with 45 mL of BHm medium was added as a negative control. Given that the strain KT2440 is not able to use mannitol as the sole C source, this condition was not tested. The Erlenmeyer flasks were incubated at 25°C under 150 rpm agitation. At each time point, 3 mL of each bacterial culture was sampled to estimate bacterial growth by measuring the OD_595nm_ and to measure the pH of the solution using a probe. Identification and quantification of the organic acids produced were performed in triplicate at the final incubation time point (i.e., 72 h). For this purpose, 6 mL of each culture was centrifuged at 7,000 × *g* for 15 min. The supernatants were then filtered at 0.22 μm (GHP Acrodisc 25 mm syringe filter; PALL) and stored at −20°C. Organic acid analyses were performed by ion chromatography with conductivity detection (ICS5000+, Thermo Scientific Corp.) associated with an analytical column (IonPac AS 11 HC, 2 × 250 mm; Thermo Scientific) according to an adapted method based on Balland et al. (50, 51). The supernatants were eluted with KOH solutions of varying concentrations (0.9–60 mM) over time (step gradient) at a flow rate of 0.38 mL·min^−1^. Synthetic organic acids were used as references (sodium formate, sodium D-gluconate, sodium butyrate, sodium pyruvate, sodium citrate tribasic, sodium oxalate, sodium propionate, sodium acetate, succinate disodium salt, DL-malate disodium salt, sodium-L-lactate, and malonate disodium salt). All standards were purchased from Inorganic Ventures, except for sodium D-gluconate (Sigma‒Aldrich) and sodium pyruvate (Fluka). The presence of D-gluconate anions in the samples was confirmed by ion detection at m/z = 194 with a single quad mass spectrometer (ISQ-EC, Thermo Scientific Corp.). The values are presented as the mean of three replicates.

### Phylogenetic and genomic analyses

For the *in silico* analyses, the genomes of a total of 80 *Pseudomonas* strains were considered, including the 10 model strains used in this study (Table S2). The additional 70 *Pseudomonas* strains were selected on the basis of the following criteria: (i) isolation mainly from the rhizosphere, (ii) availability of genetic evidence supporting their ability to weather minerals through the acidification-driven MWe mechanism, (iii) close phylogenetic relatedness to strain PML3(3), and (iv) availability of the genome sequence on NCBI. Strain selection was performed using the Type Strain Genome Server (TYGS) (52) and on the basis of the study of Švec et al. (53), which first described the *P. spealei* species to which strain PML3(3) belongs. To better understand the evolutionary relationships and the diversity of the strains selected for this study, we generated an organismal tree. For this purpose, we searched the 347 BUSCO markers from the gammaproteobacterial odb12 lineage data set (14 May 2025 release) in the proteomes using BUSCO (54). Among the 347 markers, 230 were found in single copies in all the proteomes. The identified protein sequence homologs in each species for each marker were aligned with MAFFT (55), and the protein alignments were concatenated into a superalignment with 230 partitions defined by each marker. To select the best-fit amino acid substitution models for each partition, we used ModelTest-NG (56). Maximum likelihood inference for our phylogenomic data set was achieved with RaxML-NG (57) using a partitioned analysis and setting –best-trees option to autoMRE, which generates bootstrap trees until they converge.

A targeted analysis was performed on genes associated with the acidification-driven MWe mechanism. The presence/absence of genes encoding the PQQ cofactor (i.e., *pqqFABCDEG*), GDH-PQQ (*gdh-PQQ*), and GADH-FAD (*gadh-FAD*) was considered. Gene detection was carried out on MicroScope MaGe (Magnifying Genomes) (58) or directly on NCBI using BLASTp (59). The following protein sequences were used as queries: (i) the genes *pqqFABCDE* encoding the PQQ system from *Pseudomonas pergaminensis* F77 (AQT96818.1, AQT97538.1, AQT96819.1, AQT96820.1, AQT96821.1, and AQT96822.1) and *pqqG* from *Pseudomonas ogarae* F113 (ACT32394.1); (ii) *gdh-PQQ* from *Pseudomonas pergaminensis* F77 (AQT92806.1); and (iii) the genes of (a) the small (ATW63844.1), (b) the large (ATW63845.1), and (c) the cytochrome C (ATW63846.1) subunits of the GADH-FAD from *Pseudomonas plecoglossicida* JUIM01. Research on twin-arginine translocation (TAT) signal-carrying proteins, which are necessary for the translocation of the small subunit of GADH-FAD from the cytoplasm to the periplasm, was performed using the predictor server TatP (60) to validate the identified GMC oxidoreductases. In addition to the direct BLASTp search, protein sequences from strains in which GDH-PQQ activity has been experimentally demonstrated to be involved in acidification-driven MWe were included in the analysis (i.e., *Pseudomonas pergaminensis* F77 [AQT92806.1] [61]; *Pseudomonas fluorescens* X [AEA48996.1] [62]; *Acinetobacter calcoaceticus* LMD 79.41 [CAA30222.1] [63]; *Enterobacter asburiae* PSI3 [AAQ93677.2] [64]; *Pantoea ananatis* SC17(0) [ADD81202.1] [65]; *Rahnella aquatilis* HX2 [AEA48996.1] [66]; and *Serratia* sp. S119 [ONK20138.1] [67]). The GDH-PQQ sequences were aligned with MAFFT v7.526 (68) with the L-INS-i accuracy-oriented method; the best substitution model was defined with ModelTest-NG v0.1.7; and a maximum likelihood inference of our phylogenetic data set was achieved with RAxML-NG 1.2.2 (57), using the model predicted by ModelTest-NG (56) and the MRE-based bootstrap convergence criterion (54–56). The GDH-PQQ of *Pantoea ananatis* SC17(0) was used as an outgroup.

A third phylogenetic tree was generated using a concatenated hybrid protein of the small, large, and cytochrome C subunits of putative GADH-FAD identified in the 80 *Pseudomonas* genomes. In addition to the direct BLASTp search, sequences of a set of GMC oxidoreductases whose activity has been demonstrated were added to the analysis. This is the case for the GADH-FAD from *Pantoea cypripedii* (O34213.1, O34214.2, and O34215.3) (69), *Gluconobacter dioxyacetonicus* IFO 3271 (BAF52626.1, BAF52627.1, and BAF52628.1) (70), *Gluconobacter frateurii* THF55 (BAH80544.1, BAH80545.1, and BAH80546.1) (71), and *Pseudomonas plecoglossicida* JUIM01 (ATW63844.1, ATW63845.1, and ATW63846.1)(72). Two homologs of GADH-FAD found in the genome of *Caballeronia mineralivorans* PML1(12), a strain known to be effective in weathering minerals, were also included (73).

### Statistical analysis

Statistical analyses were performed in R software (74) using the tidyverse package. Differences between the means of triplicate (growth, pH, halo diameter, colony diameter, and organic acid production) or quadruplicate (iron detection, pH) samples were analyzed using ANOVA followed by Tukey’s HSD tests, with a *P* value < 0.05. The data are presented as the mean ± standard deviation, and the different letters (e.g., a, b, c, and d) indicate significant differences among means. Linear regression analysis was used to determine potential correlations between measured data.

## RESULTS

### Metabolic characterization of the *Pseudomonas* strains

**Fig 1:**
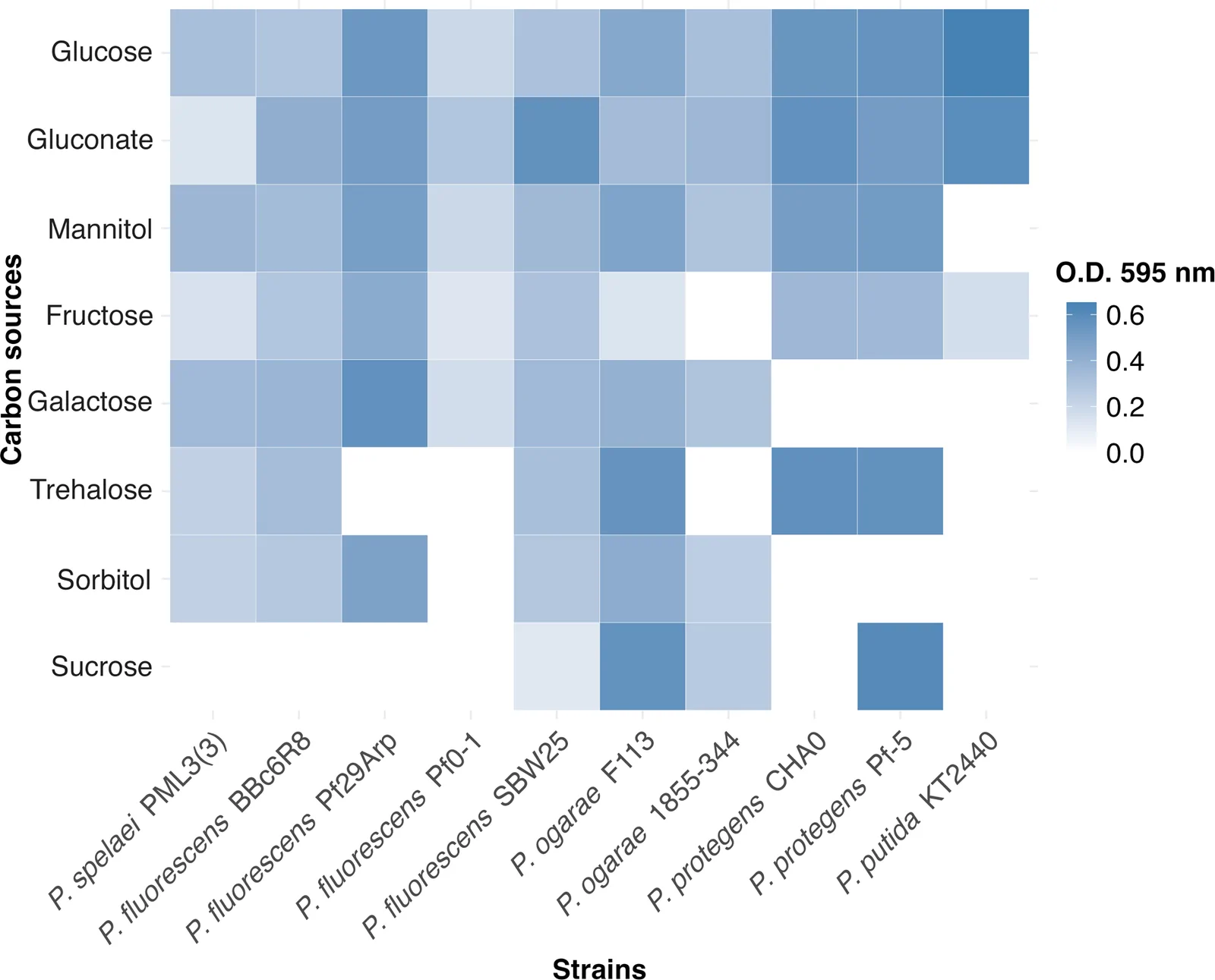
Heatmap visualization of the assimilation ability of eight carbon sources commonly found in the mycorrhizosphere by the collection of *Pseudomonas*. Substrate utilization was assessed using BIOLOG GEN III microplates by measuring OD_595_nm after 2 days of incubation. Color intensity represents the utilization level of each substrate according to the legend.

As we hypothesized that the type of carbon source influences bacterial MWe, the metabolic profile of each strain was determined using BIOLOG GEN III microplates to identify carbon sources suitable for the MWe assays. Overall, the BIOLOG metabolic profiles revealed substantial differences in carbon source utilization among the strains (Fig. S1). As expected, representatives of the same species clustered together (e.g., *P. ogarae* F113 and 1855-344 and *P. protegens* Pf-5 and CHA0), whereas greater variability was observed among the *P. fluorescens* strains. Among the substrates utilized, eight carbon sources commonly found in plant root exudates, fungal exudates, or biomass, or generated during organic matter decomposition were selected for subsequent MWe assays: glucose, galactose, trehalose, fructose, sucrose, mannitol, sorbitol, and gluconate. A heatmap analysis (Fig. 1) revealed that glucose and gluconate were utilized by all strains (10/10), and mannitol and fructose were utilized by most strains (9/10), whereas sucrose was utilized by only four strains (4/10). Carbon source utilization also varied markedly among strains. *P. fluorescens* SBW25 and *P. ogarae* F113 utilized all eight carbon sources, whereas *P. spelaei* PML3(3) and *P. fluorescens* BBc6R8 utilized all except sucrose. In contrast, *P. putida* KT2440 utilized only glucose, gluconate, and fructose.

### Effects of glucose on the growth and effectiveness in weathering of *Pseudomonas* strains

As glucose is usually the carbon substrate associated with the greatest effectiveness in weathering minerals, a first screening was performed using this substrate. After a 3-day incubation in liquid BHm medium supplemented with glucose and in the absence of biotite, compared with the abiotic control, all the strains significantly acidified the solution (pH_strains_ ≤ 4.5 ± 0.0 vs pH_abiotic_ = 6.1; *P* < 0.05). (Table 1). The lowest pH values (3.7) were measured for *P. spelaei* PML3(3), *P. fluorescens* SBW25, and *P. protegens* Pf-5. Notably, acidification was associated with weak growth (low OD_595_nm), except for the strains *P. fluorescens* BBc6R8, *P. ogarae* F113, and *P. ogarae* 1855-344, for which the OD_595_nm reached 0.3 ± 0.0 to 0.5 ± 0.2 (Table 1). After a 14-day incubation period on TCP plates supplemented with glucose, all the strains formed solubilization halos (Table 1). Significant differences were observed among the strains, with halo diameters ranging from 0.9 ± 0.0 cm for *P. putida* KT2440 to 1.2 ± 0.1 cm for *P. spelaei* PML3(3) and reaching a maximum of 1.6 ± 0.0 cm for *P. fluorescens* BBc6R8. Notably, the ability to weather Ca_3_(PO_4_)_2_ was often associated with very limited growth, as indicated by the colony diameter and apparent colony biomass. With respect to biotite weathering, a significant increase in iron release was observed for 8/10 of the *Pseudomonas* strains compared with that of the abiotic control, except for the two *P. ogarae* strains (Table 1). Among the tested strains, *P. fluorescens* BBc6R8 and *P. spelaei* PML3(3) were the most effective in releasing iron in solution ([Fe] = 0.8 ± 0.1 mg·L^−1^ for both). The most effective MWe strains also generated the greatest acidification of the solution (e.g., pH _PML3(3)_ = 3.6 ± 0.1 vs pH_F113_ = 4.4 ± 0.1 or pH_1855-344_ = 4.7 ± 0.0), highlighting the strong relationship between the [Fe] in solution and the pH (y = −1.39x + 4.77, *R*^2^ = 0.77, *P* < 0.0001). A comparison of the values obtained for each strain with reference curves corresponding to acidolysis- or acidocomplexolysis-driven MWe mechanisms confirmed that acidification was the predominant mechanism used by *Pseudomonas* strains for MWe under our experimental conditions.

**TABLE 1:** Physiological and MWe abilities of the collection of *Pseudomonas* strains across 10 carbon sources commonly found in the mycorrhizosphere^*a*^

Carbon source and strain	Solubilization and pH assays					Physiological assays	
	TCP		BHm with biotite		BHm without biotite		Biolog
	ØH (cm)	ØC (cm)	[Fe] (mg·L^−1^)	pH	pH	(OD_595nm_)	(OD_595nm_)
Glucose							
Abiotic	0.0^a^ ± 0.0	0.0^a^ ± 0.0	0.1^a^ ± 0.1	5.8^h^ ± 0.1	6.1^f^ ± 0.0	0.0^a^ ± 0.0	n.d.
PN3(3)	n.d.	n.d.	0.1^a^ ± 0.1	4.6^fg^ ± 0.0	n.d.	n.d.	n.d.
PML1(12)	n.d.	n.d.	1.2^e^ ± 0.1	3.2^a^ ± 0.1	n.d.	n.d.	n.d.
PML3(3)	1.2^cd^ ± 0.1	0.4^b^ ± 0.1	0.8^cd^ ± 0.1	3.6^bc^ ± 0.1	3.7^ab^ ± 0.1	0.2^bc^ ± 0.0	+
BBc6R8	1.6^f^ ± 0.0	0.5^bc^ ± 0.1	0.8^d^ ± 0.1	3.5^b^ ± 0.1	3.9^bc^ ± 0.2	0.5^e^ ± 0.2	+
Pf29Arp	1.0^bc^ ± 0.1	0.5^bc^ ± 0.1	0.7^bcd^ ± 0.1	3.7^bc^ ± 0.1	3.9^c^ ± 0.0	0.1^abc^ ± 0.1	+
Pf0-1	1.2^cd^ ± 0.2	0.6^cd^ ± 0.0	0.5^b^ ± 0.1	4.4^e^ ± 0.1	4.2^d^ ± 0.0	0.1^ab^ ± 0.0	+
SBW25	1.5^ef^ ± 0.2	0.5^bc^ ± 0.1	0.6^bc^ ± 0.1	3.7^ef^ ± 0.0	3.7^a^ ± 0.0	0.1^ab^ ± 0.0	+
F113	0.9^b^ ± 0.1	0.4^b^ ± 0.0	0.2^a^ ± 0.1	4.4^ef^ ± 0.1	4.3^d^ ± 0.0	0.4^de^ ± 0.0	+
1855-344	1.4^de^ ± 0.1	0.4^b^ ± 0.1	0.2^a^ ± 0.0	4.7^g^ ± 0.0	4.5^e^ ± 0.0	0.3^cd^ ± 0.0	+
CHA0	1.0^bc^ ± 0.1	0.4^b^ ± 0.1	0.7^cd^ ± 0.1	4.0^d^ ± 0.2	3.9^c^ ± 0.0	0.1^abc^ ± 0.1	+
Pf-5	1.0^bc^ ± 0.1	0.4^b^ ± 0.1	0.6^bcd^ ± 0.1	3.8^c^ ± 0.1	3.7^ab^ ± 0.1	0.1^ab^ ± 0.0	+
KT2440	0.9^b^ ± 0.0	0.7^d^ ± 0.1	0.5^b^ ± 0.1	4.4^ef^ ± 0.2	4.0^c^ ± 0.0	0.1^ab^ ± 0.0	+
Xylose							
Abiotic	0.0^a^ ± 0.0	0.0^a^ ± 0.0	0.1^a^ ± 0.1	5.8^f^ ± 0.1	6.1^g^ ± 0.0	0.0^a^ ± 0.0	n.d.
PN3(3)	n.d.	n.d.	0.1^a^ ± 0.1	5.6^f^ ± 0.4	n.d.	n.d.	n.d.
PML1(12)	n.d.	n.d.	0.1^a^ ± 0.1	5.7^f^ ± 0.3	n.d.	n.d.	n.d.
PML3(3)	1.2^bcd^ ± 0.1	0.4^b^ ± 0.0	0.5^cd^ ± 0.1	4.0^abc^ ± 0.0	3.9^cd^ ± 0.0	0.1^b^ ± 0.0	n.d.
BBc6R8	2.2^e^ ± 0.3	0.5^b^ ± 0.1	0.7^de^ ± 0.1	3.7^a^ ± 0.0	3.6^a^ ± 0.2	0.4^e^ ± 0.0	n.d.
Pf29Arp	1.5^d^ ± 0.1	0.4^b^ ± 0.1	0.6^cde^ ± 0.1	3.9^ab^ ± 0.1	3.7^ab^ ± 0.0	0.2^c^ ± 0.0	n.d.
Pf0-1	0.9^b^ ± 0.1	0.4^b^ ± 0.0	0.7^e^ ± 0.1	4.3^cd^ ± 0.1	4.1^ef^ ± 0.0	0.2^c^ ± 0.0	n.d.
SBW25	1.7^de^ ± 0.3	0.5^b^ ± 0.1	0.6^cde^ ± 0.1	3.9^a^ ± 0.1	3.8^bc^ ± 0.0	0.2^c^ ± 0.0	n.d.
F113	1.3^bcd^ ± 0.3	0.5^b^ ± 0.1	0.2^ab^ ± 0.1	4.6^d^ ± 0.1	4.1^ef^ ± 0.0	0.3^d^ ± 0.0	n.d.
1855-344	1.4^bcd^ ± 0.2	0.4^b^ ± 0.1	0.1^a^ ± 0.1	5.2^e^ ± 0.1	4.2^f^ ± 0.1	0.4^e^ ± 0.0	n.d.
CHA0	1.5^d^ ± 0.2	0.4^b^ ± 0.1	0.5^c^ ± 0.1	3.9^a^ ± 0.0	3.8^abc^ ± 0.1	0.1^b^ ± 0.0	n.d.
Pf-5	1.5^cd^ ± 0.2	0.4^b^ ± 0.0	0.4^bc^ ± 0.1	3.9^bd^ ± 0.1	3.8^bcd^ ± 0.1	0.1^b^ ± 0.0	n.d.
KT2440	1.0^bc^ ± 0.0	0.4^b^ ± 0.1	0.5^cd^ ± 0.1	4.3^bcd^ ± 0.1	4.0^de^ ± 0.0	0.1^b^ ± 0.0	n.d.
Galactose							
Abiotic	0.0^a^ ± 0.0	0.0^a^ ± 0.0	0.1^a^ ± 0.1	5.8^i^ ± 0.1	6.1^g^ ± 0.0	0.0^a^ ± 0.0	n.d.
PN3(3)	n.d.	n.d.	0.1^a^ ± 0.1	4.7^ef^ ± 0.1	n.d.	n.d.	n.d.
PML1(12)	n.d.	n.d.	0.7^f^ ± 0.1	3.6^a^ ± 0.1	n.d.	n.d.	n.d.
PML3(3)	0.9^c^ ± 0.1	0.4^b^ ± 0.0	1.0^g^ ± 0.1	3.7^ab^ ± 0.1	3.7^a^ ± 0.0	0.1^b^ ± 0.0	+
BBc6R8	1.4^d^ ± 0.1	0.5^bc^ ± 0.0	0.5^ef^ ± 0.1	4.0^bc^ ± 0.1	3.8^a^ ± 0.1	0.5^f^ ± 0.1	+
Pf29Arp	0.9^c^ ± 0.1	0.5^bc^ ± 0.0	0.6^f^ ± 0.1	3.8^b^ ± 0.1	4.0^bc^ ± 0.1	0.3^d^ ± 0.0	+
Pf0-1	0.5^b^ ± 0.1	0.8^e^ ± 0.1	0.5^ef^ ± 0.2	4.5^e^ ± 0.1	4.5^de^ ± 0.1	0.2^c^ ± 0.0	+
SBW25	0.9^c^ ± 0.1	0.5^bc^ ± 0.0	0.5^ef^ ± 0.1	3.9^b^ ± 0.1	3.9^ab^ ± 0.1	0.1^b^ ± 0.0	+
F113	0.5^b^ ± 0.1	0.5^cd^ ± 0.1	0.1^ab^ ± 0.1	5.0^g^ ± 0.2	4.4^d^ ± 0.1	0.3^d^ ± 0.0	+
1855-344	0.6^b^ ± 0.1	0.6^d^ ± 0.1	0.2^abc^ ± 0.1	5.4^h^ ± 0.0	4.8^f^ ± 0.1	0.4^e^ ± 0.0	+
CHA0	1.0^c^ ± 0.1	0.4^b^ ± 0.0	0.4^cde^ ± 0.1	4.2^cd^ ± 0.0	4.1^c^ ± 0.0	0.1^b^ ± 0.0	RL
Pf-5	0.9^c^ ± 0.1	0.4^bc^ ± 0.1	0.3^bcd^ ± 0.0	4.3^d^ ± 0.1	4.1^c^ ± 0.0	0.1^ab^ ± 0.1	RL
KT2440	0.6^b^ ± 0.0	0.5^bc^ ± 0.0	0.4^de^ ± 0.1	4.8^f^ ± 0.1	4.6^e^ ± 0.1	0.1^b^ ± 0.0	RL
Fructose							
Abiotic	0.0^a^ ± 0.0	0.0^a^ ± 0.0	0.1^a^ ± 0.1	5.8^f^ ± 0.1	6.0^h^ ± 0.1	0.0^a^ ± 0.0	n.d.
PN3(3)	n.d.	n.d.	0.5^bc^ ± 0.1	4.9^d^ ± 0.2	n.d.	n.d.	n.d.
PML1(12)	n.d.	n.d.	0.2^a^ ± 0.1	4.7^cd^ ± 0.2	n.d.	n.d.	n.d.
PML3(3)	0.7^cd^ ± 0.0	0.7^cde^ ± 0.1	0.8^e^ ± 0.1	3.9^a^ ± 0.1	3.6^a^ ± 0.0	0.1^b^ ± 0.0	+
BBc6R8	0.8^d^ ± 0.1	0.7^cde^ ± 0.1	0.7^de^ ± 0.1	4.1^a^ ± 0.1	3.7^ab^ ± 0.0	0.3^d^ ± 0.1	+
Pf29Arp	0.7^cd^ ± 0.1	0.7^bcd^ ± 0.1	0.2^a^ ± 0.1	6.2^g^ ± 0.1	3.9^c^ ± 0.1	0.3^d^ ± 0.0	+
Pf0-1	0.6^bc^ ± 0.0	0.7^cde^ ± 0.0	0.5^b^ ± 0.1	4.4^b^ ± 0.0	4.5^e^ ± 0.1	0.2^c^ ± 0.0	RL
SBW25	0.9^e^ ± 0.1	0.8^de^ ± 0.1	0.2^a^ ± 0.0	4.4^b^ ± 0.2	4.1^d^ ± 0.0	0.1^b^ ± 0.0	+
F113	0.6^c^ ± 0.1	0.6^bc^ ± 0.0	0.2^a^ ± 0.1	5.5^e^ ± 0.1	4.7^f^ ± 0.0	0.3^cd^ ± 0.1	RL
1855-344	0.7^cd^ ± 0.0	0.6^bc^ ± 0.0	0.1^a^ ± 0.0	5.6^ef^ ± 0.1	5.1^g^ ± 0.1	0.3^d^ ± 0.0	RL
CHA0	0.9^e^ ± 0.1	0.8^e^ ± 0.1	0.6^bcd^ ± 0.1	3.8^a^ ± 0.0	3.8^bc^ ± 0.0	0.2^c^ ± 0.0	+
Pf-5	0.9^e^ ± 0.0	0.8^de^ ± 0.0	0.6^bcde^ ± 0.1	3.9^a^ ± 0.1	3.8^bc^ ± 0.0	0.2^c^ ± 0.0	+
KT2440	0.5^b^ ± 0.0	0.5^b^ ± 0.1	0.7^cde^ ± 0.1	4.5^bc^ ± 0.2	4.6^ef^ ± 0.0	0.2^c^ ± 0.0	+
Mannitol							
Abiotic	0.0^a^ ± 0.0	0.0^a^ ± 0.0	0.1^ab^ ± 0.1	5.8^g^ ± 0.1	6.1^g^ ± 0.0	0.0^a^ ± 0.0	n.d.
PN3(3)	n.d.	n.d.	0.0^a^ ± 0.0	6.2^h^ ± 0.1	n.d.	n.d.	n.d.
PML1(12)	n.d.	n.d.	0.3^abc^ ± 0.1	4.2^cde^ ± 0.1	n.d.	n.d.	n.d.
PML3(3)	0.8^cd^ ± 0.0	0.8^d^ ± 0.1	1.3^d^ ± 0.2	3.8^a^ ± 0.1	3.7^a^ ± 0.1	0.2^bcd^ ± 0.1	+
BBc6R8	0.8^c^ ± 0.1	0.7^c^ ± 0.0	0.4^bc^ ± 0.2	4.9^f^ ± 0.1	3.7^a^ ± 0.1	0.4^e^ ± 0.0	+
Pf29Arp	0.7^bc^ ± 0.0	0.7^c^ ± 0.0	0.4^bc^ ± 0.2	4.3^de^ ± 0.3	4.0^b^ ± 0.0	0.3^cd^ ± 0.1	+
Pf0-1	0.6^b^ ± 0.1	0.9^d^ ± 0.1	0.5^c^ ± 0.2	4.4^e^ ± 0.0	4.6^d^ ± 0.1	0.2^bcd^ ± 0.0	+
SBW25	0.9^de^ ± 0.1	0.9^d^ ± 0.1	0.6^c^ ± 0.1	3.9^ab^ ± 0.1	4.2^c^ ± 0.0	0.2^bcd^ ± 0.0	+
F113	0.6^b^ ± 0.1	0.5^b^ ± 0.0	0.1^ab^ ± 0.1	5.6^g^ ± 0.1	4.8^e^ ± 0.1	0.2^cd^ ± 0.1	+
1855-344	0.6^b^ ± 0.1	0.7^b^ ± 0.0	0.1^ab^ ± 0.1	5.6^g^ ± 0.1	5.3^f^ ± 0.1	0.3^de^ ± 0.0	+
CHA0	0.8^cd^ ± 0.0	0.8^cd^ ± 0.0	0.6^c^ ± 0.1	4.0^abc^ ± 0.1	4.0^b^ ± 0.1	0.3^de^ ± 0.0	+
Pf-5	1.0^e^ ± 0.1	0.9^d^ ± 0.0	0.5^c^ ± 0.1	4.1^bcd^ ± 0.1	4.2^c^ ± 0.1	0.2^cd^ ± 0.1	+
KT2440	0.0^a^ ± 0.0	0.4^b^ ± 0.0	0.0^a^ ± 0.0	5.7^g^ ± 0.1	6.0^g^ ± 0.0	0.1^ab^ ± 0.0	RL
Sorbitol							
Abiotic	0.0^a^ ± 0.0	0.0^a^ ± 0.0	0.1^a^ ± 0.1	5.8^cd^ ± 0.1	6.1^e^ ± 0.0	0.0^a^ ± 0.0	n.d.
PN3(3)	n.d.	n.d.	0.0^a^ ± 0.0	6.2^e^ ± 0.1	n.d.	n.d.	n.d.
PML1(12)	n.d.	n.d.	0.5^cd^ ± 0.1	4.2^a^ ± 0.1	n.d.	n.d.	n.d.
PML3(3)	0.8^de^ ± 0.0	0.9^e^ ± 0.1	1.1^e^ ± 0.3	3.9^a^ ± 0.1	3.7^a^ ± 0.1	0.2^c^ ± 0.0	+
BBc6R8	0.7^cde^ ± 0.1	0.6^c^ ± 0.1	0.8^d^ ± 0.1	4.1^a^ ± 0.3	4.0^b^ ± 0.0	0.3^d^ ± 0.1	+
Pf29Arp	0.7^cd^ ± 0.0	0.7^cd^ ± 0.1	0.4^bc^ ± 0.2	4.7^b^ ± 0.1	4.1^b^ ± 0.0	0.1^b^ ± 0.0	+
Pf0-1	0.0^a^ ± 0.0	0.5^b^ ± 0.0	0.0^a^ ± 0.1	5.9^de^ ± 0.2	6.1^e^ ± 0.0	0.3^d^ ± 0.0	RL
SBW25	0.8^e^ ± 0.1	0.8^de^ ± 0.0	0.4^c^ ± 0.1	4.7^b^ ± 0.1	4.1^b^ ± 0.0	0.2^c^ ± 0.0	+
F113	0.6^b^ ± 0.1	0.5^b^ ± 0.0	0.1^a^ ± 0.1	5.7^cd^ ± 0.2	4.7^c^ ± 0.1	0.4^d^ ± 0.1	+
1855-344	0.7^bc^ ± 0.1	0.7^c^ ± 0.1	0.1^a^ ± 0.1	5.6^cd^ ± 0.1	4.9^d^ ± 0.1	0.3^d^ ± 0.0	+
CHA0	0.0^a^ ± 0.0	0.5^b^ ± 0.0	0.0^a^ ± 0.1	5.7^cd^ ± 0.1	6.0^e^ ± 0.0	0.1^b^ ± 0.0	RL
Pf-5	0.0^a^ ± 0.0	0.5^b^ ± 0.1	0.0^a^ ± 0.1	5.7^cd^ ± 0.2	6.0^e^ ± 0.1	0.1^b^ ± 0.0	RL
KT2440	0.0^a^ ± 0.0	0.5^b^ ± 0.0	0.1^ab^ ± 0.0	5.5^c^ ± 0.0	6.1^e^ ± 0.0	0.1^b^ ± 0.0	RL
Trehalose							
Abiotic	0.0^a^ ± 0.0	0.0^a^ ± 0.0	0.1^a^ ± 0.1	5.8^f^ ± 0.1	6.1^d^ ± 0.0	0.0^a^ ± 0.1	n.d.
PN3(3)	n.d.	n.d.	0.0^a^ ± 0.0	6.1^g^ ± 0.1	n.d.	n.d.	n.d.
PML1(12)	n.d.	n.d.	0.0^a^ ± 0.0	5.6^e^ ± 0.1	n.d.	n.d.	n.d.
PML3(3)	0.8^c^ ± 0.0	0.7^d^ ± 0.1	1.1^d^ ± 0.2	3.8^a^ ± 0.0	4.2^b^ ± 0.1	0.2^c^ ± 0.0	+
BBc6R8	0.8^c^ ± 0.0	0.6^cd^ ± 0.1	0.4^c^ ± 0.1	3.8^ab^ ± 0.1	3.9^a^ ± 0.1	0.2^bc^ ± 0.1	+
Pf29Arp	0.0^a^ ± 0.0	0.4^b^ ± 0.1	0.0^a^ ± 0.0	5.9^f^ ± 0.1	5.9^d^ ± 0.1	0.1^ab^ ± 0.0	RL
Pf0-1	0.0^a^ ± 0.0	0.5^bc^ ± 0.0	0.0^a^ ± 0.1	5.8^f^ ± 0.0	6.1^d^ ± 0.0	0.3^d^ ± 0.0	RL
SBW25	0.9^c^ ± 0.1	0.7^d^ ± 0.1	0.3^bc^ ± 0.1	3.8^a^ ± 0.0	4.2^b^ ± 0.1	0.1^ab^ ± 0.0	+
F113	1.1^d^ ± 0.1	0.5^bc^ ± 0.0	0.2^ab^ ± 0.1	5.0^d^ ± 0.1	4.6^c^ ± 0.1	0.4^e^ ± 0.0	+
1855-344	0.6^b^ ± 0.1	0.5^b^ ± 0.1	0.0^a^ ± 0.1	5.6^e^ ± 0.1	6.1^d^ ± 0.0	0.2^c^ ± 0.0	RL
CHA0	0.8^c^ ± 0.1	0.7^d^ ± 0.1	0.4^c^ ± 0.1	4.1^bc^ ± 0.1	4.1^b^ ± 0.1	0.1^ab^ ± 0.0	+
Pf-5	0.8^c^ ± 0.0	0.7^d^ ± 0.1	0.4^c^ ± 0.1	4.1^c^ ± 0.1	4.2^b^ ± 0.1	0.1^ab^ ± 0.0	+
KT2440	0.0^a^ ± 0.0	0.5^bc^ ± 0.0	0.0^a^ ± 0.1	5.8^ef^ ± 0.2	6.1^d^ ± 0.0	0.1^ab^ ± 0.0	RL
Sucrose							
Abiotic	0.0^a^ ± 0.0	0.0^a^ ± 0.0	0.1^a^ ± 0.1	5.8^de^ ± 0.1	6.1^f^ ± 0.0	0.0^a^ ± 0.0	n.d.
PN3(3)	n.d.	n.d.	0.0^a^ ± 0.0	5.8^de^ ± 0.2	n.d.	n.d.	n.d.
PML1(12)	n.d.	n.d.	0.1^a^ ± 0.1	5.6^bcd^ ± 0.1	n.d.	n.d.	n.d.
PML3(3)	0.0^a^ ± 0.0	0.4^b^ ± 0.0	0.0^a^ ± 0.1	5.8^e^ ± 0.1	5.9^d^ ± 0.1	0.1^ab^ ± 0.0	RL
BBc6R8	0.0^a^ ± 0.0	0.5^b^ ± 0.0	0.0^a^ ± 0.0	6.1^f^ ± 0.1	6.0^de^ ± 0.1	0.2^b^ ± 0.0	RL
Pf29Arp	0.0^a^ ± 0.0	0.4^b^ ± 0.1	0.0^a^ ± 0.0	5.9^ef^ ± 0.1	6.0^def^ ± 0.0	0.1^b^ ± 0.1	RL
Pf0-1	0.0^a^ ± 0.0	0.5^b^ ± 0.0	0.0^a^ ± 0.0	5.8^de^ ± 0.1	6.1^f^ ± 0.0	0.2^b^ ± 0.1	RL
SBW25	1.0^e^ ± 0.1	0.5^b^ ± 0.0	0.0^a^ ± 0.0	5.7^de^ ± 0.1	4.8^ab^ ± 0.1	0.4^c^ ± 0.0	+
F113	0.6^b^ ± 0.0	0.5^b^ ± 0.0	0.1^a^ ± 0.1	5.4^bc^ ± 0.1	5.0^c^ ± 0.1	0.2^b^ ± 0.0	+
1855-344	0.7^c^ ± 0.0	0.6^c^ ± 0.1	0.1^a^ ± 0.0	5.4^b^ ± 0.1	4.9^b^ ± 0.0	0.3^c^ ± 0.1	+
CHA0	0.0^a^ ± 0.0	0.4^b^ ± 0.1	0.0^a^ ± 0.0	5.7^de^ ± 0.1	6.0^def^ ± 0.0	0.1^ab^ ± 0.0	RL
Pf-5	0.9^d^ ± 0.0	0.7^c^ ± 0.1	0.4^b^ ± 0.1	4.4^a^ ± 0.2	4.7^a^ ± 0.1	0.1^b^ ± 0.1	+
KT2440	0.0^a^ ± 0.0	0.5^b^ ± 0.0	0.0^a^ ± 0.1	5.7^cde^ ± 0.1	6.1^ef^ ± 0.1	0.1^ab^ ± 0.0	RL
Succinate							
Abiotic	0.0^a^ ± 0.0	0.0^a^ ± 0.0	0.1 (0.1)	5.8	6.1^a^ ± 0.0	0.0^a^ ± 0.0	n.d.
PN3(3)	n.d.	n.d.	n.d.	n.d.	n.d.	n.d.	n.d.
PML1(12)	n.d.	n.d.	n.d.	n.d.	n.d.	n.d.	n.d.
PML3(3)	0.0^a^ ± 0.0	1.0^cd^ ± 0.1	n.d.	n.d.	6.1^a^ ± 0.0	0.5^c^ ± 0.0	n.d.
BBc6R8	0.0^a^ ± 0.0	1.2^d^ ± 0.1	n.d.	n.d.	6.1^a^ ± 0.0	0.7^d^ ± 0.0	n.d.
Pf29Arp	0.0^a^ ± 0.0	1.0^cd^ ± 0.1	n.d.	n.d.	6.1^a^ ± 0.0	0.8^e^ ± 0.1	n.d.
Pf0-1	0.0^a^ ± 0.0	0.7^b^ ± 0.0	n.d.	n.d.	6.2^b^ ± 0.0	0.5^c^ ± 0.0	n.d.
SBW25	0.0^a^ ± 0.0	0.9^c^ ± 0.1	n.d.	n.d.	6.2^b^ ± 0.0	0.8^e^ ± 0.0	n.d.
F113	0.0^a^ ± 0.0	0.8^b^ ± 0.1	n.d.	n.d.	6.2^b^ ± 0.0	1.0^g^ ± 0.0	n.d.
1855-344	0.0^a^ ± 0.0	0.7^b^ ± 0.1	n.d.	n.d.	6.2^b^ ± 0.0	0.7^d^ ± 0.0	n.d.
CHA0	0.0^a^ ± 0.0	1.1^d^ ± 0.0	n.d.	n.d.	6.1^a^ ± 0.0	0.2^b^ ± 0.0	n.d.
Pf-5	0.0^a^ ± 0.0	1.1^d^ ± 0.1	n.d.	n.d.	6.1^a^ ± 0.0	0.2^b^ ± 0.0	n.d.
KT2440	0.0^a^ ± 0.0	0.9^c^ ± 0.1	n.d.	n.d.	6.2^b^ ± 0.0	0.9^f^ ± 0.0	n.d.
Gluconate							
Abiotic	0.0^a^ ± 0.0	0.0^a^ ± 0.0	0.1 (0.1)	5.8	6.1^a^ ± 0.0	0.1^a^ ± 0.1	n.d.
PN3(3)	n.d.	n.d.	n.d.	n.d.	n.d.	n.d.	n.d.
PML1(12)	n.d.	n.d.	n.d.	n.d.	n.d.	n.d.	n.d.
PML3(3)	0.0^a^ ± 0.0	1.0^de^ ± 0.1	n.d.	n.d.	6.1^a^ ± 0.0	0.7^e^ ± 0.0	RL
BBc6R8	0.0^a^ ± 0.0	1.3^g^ ± 0.1	n.d.	n.d.	6.1^a^ ± 0.0	0.3^b^ ± 0.0	+
Pf29Arp	0.0^a^ ± 0.0	1.2^ef^ ± 0.1	n.d.	n.d.	6.1^a^ ± 0.0	0.4^c^ ± 0.0	+
Pf0-1	0.0^a^ ± 0.0	0.9^bc^ ± 0.1	n.d.	n.d.	6.2^b^ ± 0.0	0.4^c^ ± 0.0	+
SBW25	0.0^a^ ± 0.0	1.4^g^ ± 0.1	n.d.	n.d.	6.2^b^ ± 0.0	0.7^e^ ± 0.0	+
F113	0.0^a^ ± 0.0	0.8^b^ ± 0.1	n.d.	n.d.	6.2^b^ ± 0.0	1.1^g^ ± 0.0	+
1855-344	0.0^a^ ± 0.0	0.7^b^ ± 0.1	n.d.	n.d.	6.2^b^ ± 0.0	0.6^d^ ± 0.0	+
CHA0	0.0^a^ ± 0.0	1.1^def^ ± 0.1	n.d.	n.d.	6.2^b^ ± 0.0	0.3^bc^ ± 0.1	+
Pf-5	0.0^a^ ± 0.0	1.2^fg^ ± 0.1	n.d.	n.d.	6.1^a^ ± 0.0	0.3^b^ ± 0.0	+
KT2440	0.0^a^ ± 0.0	1.0^cd^ ± 0.0	n.d.	n.d.	6.2^b^ ± 0.0	1.0^f^ ± 0.0	+

^*a*^ Strains PML1(12) and PN3(3) were used as positive and negative controls, respectively. For the TCP biotest, the halo and colony diameters refer to ØH and ØC, respectively. For the biotite weathering assay, the concentration of iron released in solution [Fe] and the pH of the solution are presented. For the incubation done with biotite, the suspension was used to determine the solution pH and the growth increase (i.e., OD_595nm_). For the BIOLOG assay, reactions with an OD_595nm_ < 0.15 were considered as limit reactions, and reactions with an OD_595nm_ ≥ 0.15 were considered as positive reactions. The abbreviation “n.d.” means not determined, “RL” means limit reaction, and “+” means positive reaction. The abiotic control in BHm + biotite was performed in the sucrose condition only, and the resulting pH and [Fe] values were used as references for comparison in the other conditions. Statistical analyses were carried out for each test using an analysis of variance (ANOVA), with statistical significance set at *P* < 0.05. Differences among means were analyzed using ANOVA followed by Tukey’s HSD test (*P* < 0.05). Data are presented as mean ± standard deviation, and different letters (e.g., a, b, c, d) indicate significant differences among means.

### Effects of different carbon sources on the growth and effectiveness in weathering of the *Pseudomonas* strains

**Fig 2:**
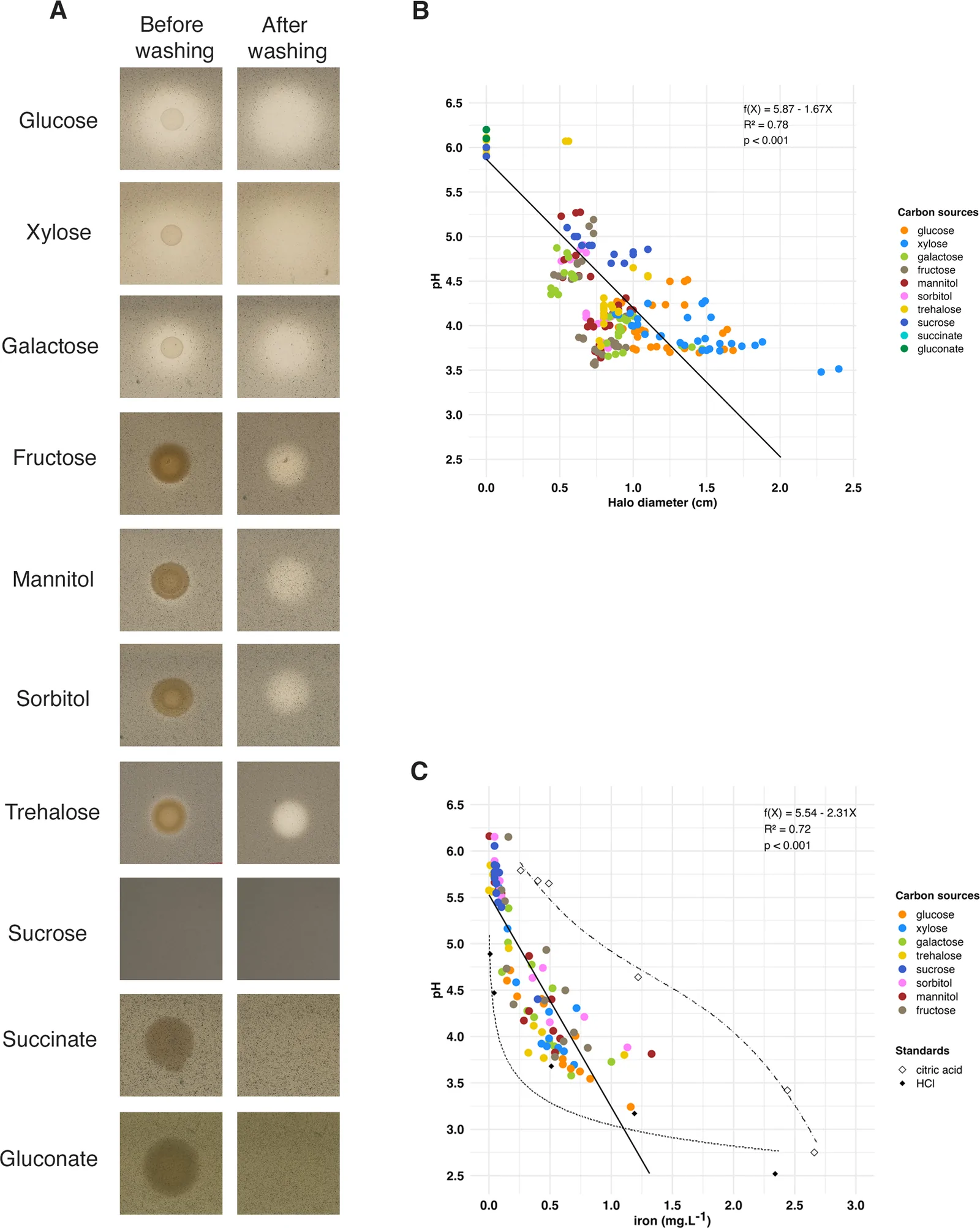
Mineral weathering ability of *P. spelaei* PML3(3) and other *Pseudomonas* strains across carbon substrates and media. (**A**) Presentation of the solubilization halos developed by strain PML3(3) on TCP. The first column shows the colony and halo (if present) around the colony. The second column shows the same conditions after washing the colonies to determine the presence/absence of solubilization below the colony. Each square measures 2 cm per side. (**B**) Relationship between solution acidification and P solubilization abilities. For the 10 *Pseudomonas* strains, the pH was measured after incubation in the BHm medium depleted in biotite, and the diameter of the solubilization halos (cm) was measured around and under colonies on TCP. Each condition is measured in triplicate. (**C**) Relationship between solution acidification and biotite weathering abilities. After incubation with biotite and different carbon sources (i.e., eight in total), the solution pH and iron concentrations were determined for each strain in *Pseudomonas* collection. The two curves correspond to the main mineral weathering mechanisms (i.e., acidocomplexolysis and acidolysis) obtained through abiotic incubation with citric acid for the acidocomplexolysis mechanism (upper line) and hydrochloric acid (HCl) for acidolysis (lower line). The coordinates of each point are the mean values of three replicates of pH and iron concentrations measured for each condition. In panels B and C, the line corresponds to the linear regression analysis performed for each data comparison. The equation of the line, coefficient of determination (*R*²), and the *P*-value (*P*) are presented in the upper right corner of panels B and C.

On the basis of the hypothesis that carbon sources released by plants or fungi could drive bacterial MWe ability, the effectiveness of the *Pseudomonas* strains in weathering minerals was assessed using additional carbon sources (i.e., xylose, galactose, trehalose, fructose, sucrose, mannitol, sorbitol, succinate, and gluconate) and the bioassays described above. All the data are presented in Table 1 and Fig. 2.

After a 3-day incubation in liquid BHm medium supplemented with different carbon sources and without biotite, distinct acidification patterns were observed among the strains (Table 1). All the strains acidified the solution when glucose, xylose, galactose, or fructose were provided as the sole carbon sources, whereas none did so with gluconate or succinate. Acidification with sorbitol, mannitol, trehalose, and sucrose was strain-dependent, with *P. fluorescens* SBW25 and *P. ogarae* F113 able to acidify with all four substrates, in contrast to *P. putida* KT2440. These results revealed that the effectiveness of acidification varied significantly among the different strains and that, for the same strain, the carbon source metabolized was an important factor. Some strains displayed a broad acidification capacity (e.g., *P. fluorescens* SBW25), whereas others were restricted to fewer substrates (e.g., *P. putida* KT2440). As observed with glucose, acidification was generally associated with limited growth, except for *P. fluorescens* BBc6R8, whose OD_595_nm values reached 0.5 ± 0.1 with galactose (pH = 3.8 ± 0.1) and 0.5 ± 0.2 with glucose (pH = 3.9 ± 0.2).

After a 14-day incubation on TCP plates, all strains, except *P. fluorescens* Pf0-1, weathered Ca_3_(PO_4_)_2_ when glucose, xylose, and galactose were provided (Table 1). The halo diameters ranged from 0.9 ± 0.1 cm for strain Pf0-1 to 2.2 ± 0.3 cm for strain BBc6R8 with xylose. For these three substrates, weathering was generally associated with very limited growth, as indicated by colony diameter and visual biomass assessment (Fig. 2A). No solubilization halos were observed with succinate or gluconate, despite strong bacterial growth (e.g., ØC_SBW25_(gluconate) = 1.4 ± 0.1 cm). The responses to fructose, mannitol, sorbitol, trehalose, and sucrose were strain-dependent, ranging from thin halos around well-developed colonies (e.g., CHA0 with fructose: ØC = 0.8 ± 0.1 cm; ØH = 0.9 ± 0.1 cm) to complete absence of growth or halo formation. Only *P. ogarae* F113 and *P. fluorescens* SBW25 produced clear halos with trehalose and sucrose. Plate washing further revealed hidden halos beneath the colonies initially showing no visible surrounding halos, except under gluconate and succinate conditions (Fig. 2A). This was the case for strain Pf0-1 using galactose, for which a halo of 0.5 ± 0.1 cm formed below a colony measuring 0.8 ± 0.1 cm.

Linear regression analyses performed on the two data sets (i.e., solubilization halos formed exclusively around colonies versus solution pH, and halos formed both around and beneath colonies versus solution pH) revealed a significant relationship between pH and halo formation. The greatest *R*² value was obtained when both peripheral and underlying halos were considered (y_around_ = −1.35x + 5.59, *R*^2^ = 0.57, *P* < 0.001; y_around&under_ = −1.67x + 5.87, *R*^2^ = 0.78, *P* < 0.001) (Fig. 2B). This relationship indicates that localized weathering occurring under the colony on TCP plates is an important indicator to consider when assessing the MWe ability of bacteria.

The 3-day incubation experiments with biotite revealed that glucose, galactose, and xylose were the carbon sources associated with the greatest number of effective MWe *Pseudomonas* strains. In contrast, *P. protegens* Pf-5 was the only strain able to weather biotite using sucrose ([Fe] = 0.4 ± 0.1 mg·L^−1^) (Fig. 2C). A comparison of the differences in the effectiveness in weathering allowed the classification of strains according to the number of substrates supporting their MWe ability (Table S3). Among them, strains PML3(3) and Pf-5 displayed the broadest substrate range for weathering biotite with 7/8 of the tested substrates. *P. spelaei* PML3(3) was the most effective in weathering for most substrates ([Fe] released in the presence of galactose, sorbitol, mannitol, fructose, and trehalose = 1.0 ± 0.1 mg·L^−1^, 1.1 ± 0.3 mg·L^−1^, 1.3 ± 0.2 mg·L^−1^, 0.8 ± 0.1 mg·L^−1^, and 1.1 ± 0.2 mg·L^−1^, respectively) (Table 1). In contrast, *P. ogarae* F113 and 1855-344 did not weather biotite with any of the tested substrates, despite showing moderate acidification for some substrates (e.g., pH_F113_ = 4.4 ± 0.1 with glucose vs pH_F113_ = 5.7 ± 0.2 with sorbitol) (Table 1).

Overall, a comparison of solution pH and iron concentrations across all carbon sources revealed that most *Pseudomonas* strains weathered biotite predominantly through an acidification-driven mechanism, as indicated by their positioning relative to the acidification and acidocomplexolysis reference curves. A linear regression analysis confirmed a significant relationship between iron concentration and solution pH (y = −2.31x + 5.54, *R*² = 0.72, *P* < 0.0001) (Fig. 2C). In contrast, when the same iron concentration data were compared with the pH values measured in the absence of biotite, the relationship was weaker (y = −1.96x + 5.26, *R*² = 0.52, *P* < 0.001). This difference suggests that the presence of biotite influenced the metabolic behavior of the bacteria and the final pH of the solution. Finally, a significant correlation was observed between the two MWe proxies (i.e., solubilization halos formed both around and beneath colonies and iron release from biotite; y = 0.78x + 0.47, *R*² = 0.25, *P* < 0.001). The relatively low *R*² value suggests that although most strains were able to weather both minerals, their relative effectiveness differed between tricalcium phosphate and biotite.

### Influence of the carbon source on the acidification dynamics of the *Pseudomonas* strains and characterization of the organic acids produced

**Fig 3:**
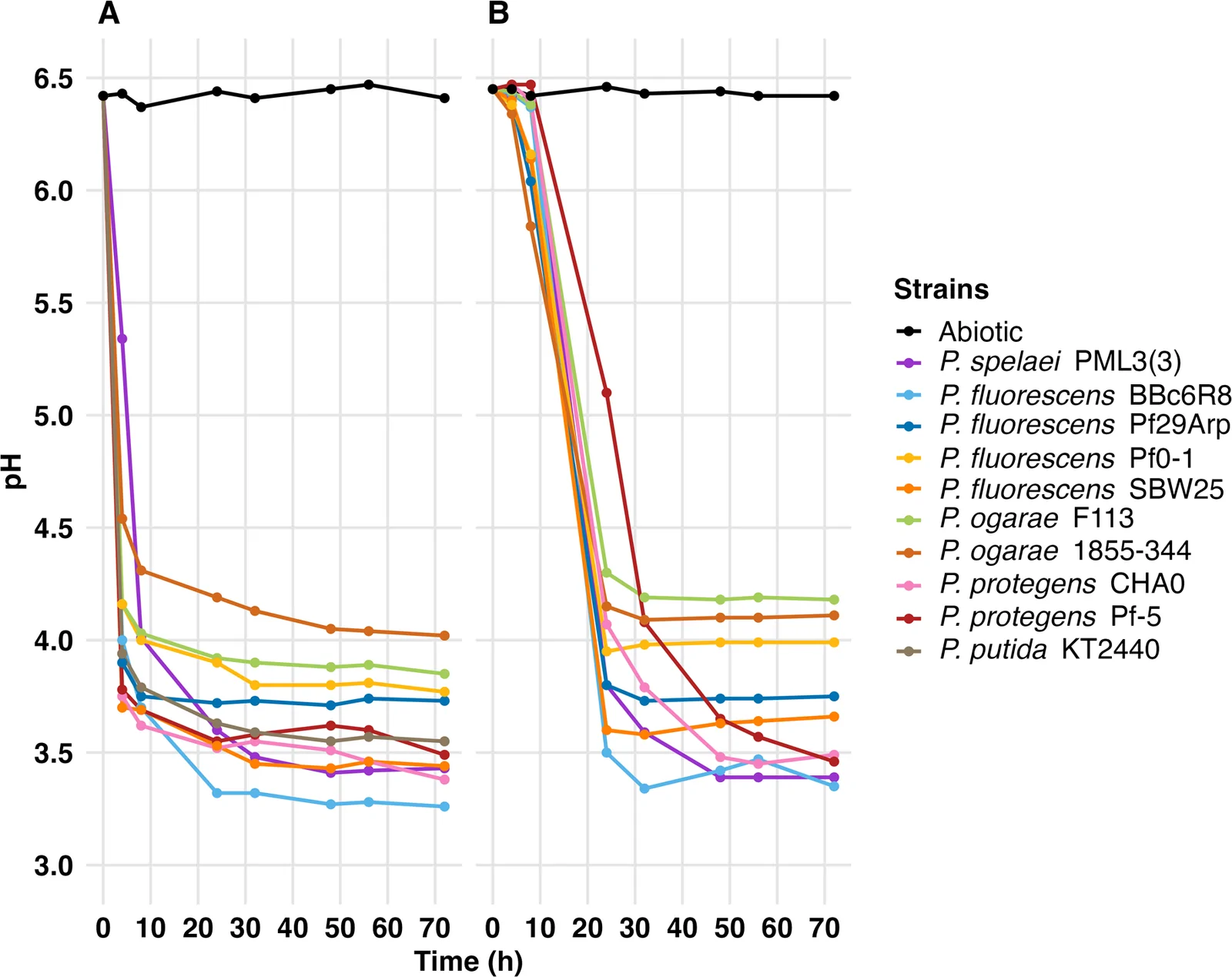
Acidification kinetics of the culture solution after incubation of the *Pseudomonas* strains in the presence of glucose (**A**) or mannitol (**B**). The pH was measured after 4, 8, 24, 32, 48, 56, and 72 h of incubation.

Despite exhibiting similar pH values after a 3-day incubation in BHm (without biotite) supplemented with glucose or mannitol, some strains displayed different phenotypes on solid TCP plates after 14 days of incubation (Fig. 2A). This is the case for the strains PML3(3), Pf29Arp, and CHA0. These strains formed a clear and very large halo around a small colony on TCP medium supplemented with glucose, whereas a thin halo formed with mannitol. In addition to the differences in incubation time (i.e., 3 days vs 14 days) and culture medium (i.e., BHm vs TCP), a carbon source-dependent delay may also explain these results. In this context, a kinetic analysis was performed for all the *Pseudomonas* strains considering glucose and mannitol. This kinetic experiment (time points: 0, 4, 8, 24, 32, 48, 56, and 72 h) did not reveal significant differences in growth, as the OD_595_nm remained low (0.1 to 0.2 after 72 h) but highlighted marked differences in the dynamics of solution acidification (Fig. 3). Indeed, the most acidic pH value was reached within the first 8 h of incubation for all the strains incubated with glucose (Table S4), ranging from 4.3 for *P. ogarae* 1855-344 to 3.6 for *P. protegens* CHA0. In contrast, during the first 8 h of incubation with mannitol, most of the strains (e.g., PML3(3), BBc6R8, CHA0, Pf-5, and F113) showed no acidification compared with the abiotic control (pH = 6.4). The pH ranged from 6.5 for *P. protegens* Pf-5 to 5.8 for *P. ogarae* 1855-344. However, after 24 h of incubation, all the strains except *P. protegens* Pf-5 (pH = 5.1, *P* < 0.05) acidified the solution below pH 4.4. Acidification reached a plateau after 8 h in BHm supplemented with glucose, whereas this plateau was reached only after 24 h with mannitol.

As organic acid production is associated with the acidification-driven MWe mechanism, a global analysis was performed after 3 days of incubation (Table S5). Chromatographic analyses revealed very low organic acid production with mannitol (total organic acid concentration < 3 mg·L⁻¹; data not shown). In contrast, in BHm supplemented with glucose, all the strains produced one or two organic acids (gluconate, lactate, and/or butyrate), with gluconate being the predominant metabolite. Notably, *P. ogarae* F113 was the only strain that produced lactate ([lactate] = 55.0 ± 5.6 mg·L⁻¹), together with the highest gluconate concentration among all the strains ([gluconate] = 44.0 ± 2.0 mg·L⁻¹). Gluconate was the sole organic acid detected in the supernatants of *P. fluorescens* SBW25, *P. ogarae* 1855-344, and *P. protegens* CHA0, whereas the remaining strains produced both gluconate and butyrate (Table S5). Finally, growth assays using gluconate as the sole carbon source revealed that all the strains, except *P. ogarae* 1855-344, were able to metabolize this substrate.

### Detection of GDH-PQQ- and GADH-FAD-dependent enzymes in the genomes of *Pseudomonas* strains

To better understand the relationships among taxonomic belonging, the genetic basis of the acidification-driven MWe mechanism, and the functional potential of the *Pseudomonas* genus, a genome analysis was performed considering a total of 80 *Pseudomonas* strains (including the 10 strains tested in our study). The creation of a multilocus tree based on the concatenated protein sequences of housekeeping genes highlighted the phylogenetic proximity of the selected strains, which can be divided into 40 different *species* (Fig. S2).

At the functional level, PQQ biosynthesis genes were identified in all 80 *Pseudomonas* genomes analyzed (Table 2). However, the organization of the *pqq* cluster and the copy number of individual *pqq* genes varied among the strains. Regardless of the *pqq* gene copy number, 35 strains harbored a *pqqFABCDE* cluster, 16 harbored a *pqqFABCDEG* cluster, 14 harbored a *pqqFBCDEG* cluster, 11 harbored a *pqqFBCD* cluster, and four strains appeared to carry a *pqqF* gene located distally from the remaining *pqq* genes. Conversely, several *pqq* genes (except *pqqF* and *pqqG*) were present in multiple copies in some genomes.

**TABLE 2:** Detail of the PQQ gene organization, key acidification-driven MWe enzyme presence, and main organic acid(s) produced for the 80 *Pseudomonas* strains^*a*^

Strain	*PQQ* cluster	GDH-PQQ	GADH-FAD	Organic acid production	Reference(s)
Collection of 10 *Pseudomonas*					
*P. spelaei* PML3(3)	FABCDE	1	1	**GA +** 2KGA	This study
*P. fluorescens* BBc6R8	FABCDE	1	1	**GA +** 2KGA	This study; Galet et al. (75)
*P. fluorescens* Pf29Arp	FBCDEG	1	0	**GA**	This study
*P. fluorescens* Pf0-1	FABCDEG	1	1	**GA + 2KGA**	This study; Miller et al. (44)
*P. fluorescens* SBW25	FABCDE	1	1	**GA +** 2KGA	This study
*P. ogarae* F113	FABCDEG/A	1	0	**GA**	This study; Miller et al. (44)
*P. ogarae* 1855-344	FBCDEG	1	0	**GA**	This study
*P. protegens* CHA0	FABCDEG/AB	1	1	**GA + 2KGA**	This study; Miller et al. (44)
*P. protegens* Pf-5	FABCDEG/AB	1	1	**GA + 2KGA**	This study; Miller et al. (44)
*P. putida* KT2440	FABCDEG/AD	1	1	**GA + 2KGA**	This study; Miller et al. (44)
*P. aeruginosa* DSM 50071	F(distal) ABCDEG	1	1	GA + 2KGA	–
*P. aeruginosa* P4	F(distal) ABCDEG	1	1	GA + 2KGA	–
*P. aeruginosa* PAO1	F(distal) ABCDEG	1	1	GA + **2KGA**	Miller et al. (44)
*P. antarctica* LMG 22709	FBCDE/C	1	1	GA + **2KGA**	Sawada et al. (76)
*P. antarctica* S101	FABCDE	1	1	GA + 2KGA	–
*P. asiatica* JP233	FABCDEG/AD	1	1	GA + **2KGA**	Yu et al. (77) This study
*P. asiatica* P1	FABCDEG/AD	1	1	GA + 2KGA	–
*P. azotoformans* LMG 21611	FABCDE	1	1	GA + 2KGA	–
*P. azotoformans* SC 14	FBCDE	1	1	GA + 2KGA	–
*P. brassicacearum* 3Re2-7	FABCDEG/A	1	0	GA	
*P. brassicacearum subsp. brassicacearum* NFM421	FBCDEG/BCDE	1	0	GA	–
*P. brenneri* DSM 15294	FBCDE	1	1	GA + 2KGA	–
*P. brenneri* Ps-23	FABCDE	1	1	GA + 2KGA	–
*P. capeferrum* PseudoSSchier:88	FBCDEG/DE and two copies of partial *pqqC*	1	1	GA + 2KGA	–
*P. capeferrum* WCS358	FBCDEG/DE and one copy of partial *pqqC*	1	1	**GA + 2KGA**	Yu et al. (78)
*P. chlororaphis* DSM 295782	FABCDEG	1	1	GA + 2KGA	–
*P. chlororaphis* O6	FBCDEG	1	1	GA + 2KGA	–
*P. edaphica* B7002	FABCDE	1	1	GA + 2KGA	–
*P. edaphica* RD25	F(distal) ABCDE	1	1	GA + 2KGA	–
*P. extremaustralis* 14-3 *substr.* 14-3b	FABCDE	1	0	GA	–
*P. extremaustralis* 1906	FBCDE	1	0	GA	–
*P. extremorientalis* FP1754	FABCDE/A	1	1	GA + 2KGA	–
*P. extremorientalis* LMG 19695	FABCDE/A	1	1	GA + 2KGA	–
*P. frederiksbergensis* 11-D3	FBCDEG/BCE	1	0	GA	–
*P. frederiksbergensis* 38F7	FBCDEG / BCE	1	1	GA + 2KGA	–
*P. gessardii* DSM 17152	FABCDE	1	1	GA + 2KGA	–
*P. gessardii* G4779	FABCDE	1	1	GA + 2KGA	–
*P. glycinae* MS586	FABCDEG	1	1	GA + 2KGA	–
*P. glycinae* XJFL-1	FABCDEG	1	1	GA + 2KGA	–
*P. gregormendelii* LMG 28632	FABCDEG/ABCE	1	0	GA	–
*P. grimontii* BIGb0428	FABCDE/BCDE	2	1	GA + 2KGA	–
*P. grimontii* D118	FABCDE/ABCDE	1	1	GA + 2KGA	–
*P. grimontii* DSM 17515	FABCDE	1	1	GA + 2KGA	–
*P. karstica* CCM 7891	FABCDE	2	2	GA + 2KGA	–
*P. kielensis* MBT-1	FABCDEG/ABCDE	1	0	GA	–
*P. kielensis* ZE23JCel16	FABCDEG/ABCDE	1	0	GA	–
*P. lactis* DSM 29167	FBCDE	1	1	GA + 2KGA	–
*P. libanensis* DSM 17149	FABCDE	1	1	GA + 2KGA	–
*P. libanensis* RIT-PI-g	FBCDE	1	1	GA + 2KGA	–
*P. lutea* CFBP13723	FABCDEG	3	1	GA	–
*P. lutea* LMG 21974	FBCDEG	2	1	GA + 2KGA	–
*P. marginalis* DSM 13124	FBCDE/two copies of *pqqA*	1	1	GA + **2KGA**	Sawada et al. (76)
*P. marginalis* DSM 18529	FABCDE/A	1	1	GA + 2KGA	–
*P. migulae* CFML 95-321	FBCDEG/BCDE	1	0	GA	–
*P. migulae* LMG 21608	FBCDEG/BCDE	1	0	GA	–
*P. mucidolens* NCTC 8068	FABCDE/A	1	0	GA	–
*P. orientalis* 8B	FBCDE	1	1	GA + 2KGA	–
*P. orientalis* L1-3-08	FBCDE	1	1	GA + 2KGA	–
*P. pergaminensis* 1008	FABCDE/A	1	2	GA + 2KGA	–
*P. pergaminensis* F77	FABCDE/A	1	1	**GA +** 2KGA	Wang et al. (59)
*P. pergaminensis* Phyllo_228	FABCDE/A	1	1	GA + 2KGA	–
*P. petroselini* MAFF 311094	FABCDE	1	1	GA + 2KGA	–
*P. proteolytica* DSM 15321	FBCDE	1	1	GA + 2KGA	–
*P. proteolytica* PA-6-9F	FABCDE	1	1	GA + 2KGA	–
*P. putida* NBRC 14164	FBCDEG	1	1	GA + 2KGA	–
*P. rhizosphaerae* CFBP8984	FABCDEG	1	1	GA + 2KGA	–
*P. rhizosphaerae* DSM 16299	FBCDEG	1	1	GA + 2KGA	–
*P. spelaei* CCM 7893	FABCDE	1	1	GA + 2KGA	–
*P. synxantha* 2-79	FABCDE	1	1	GA + 2KGA	–
*P. synxantha* NCTC 10696	FABCDE	1	1	GA + 2KGA	–
*P. synxantha* R6-28-08	FABCDE	1	1	GA + 2KGA	–
*P. tolaasii* FP2293	FABCDE	1	1	GA + 2KGA	–
*P. tolaasii* NCPPB 2192	FABCDE	1	1	GA + 2KGA	–
*P. trivialis* IHBB745	FABCDE	1	2	**GA + 2KGA**	Vyas et al. (79)
*P. trivialis* LMG 21464	FBCDE	1	1	GA + **2KGA**	Sawada et al. (76)
*P. yamanorum* IPO3781	FABCDE	1	1	GA + 2KGA	–
*P. yamanorum* LMG 27247	FABCDE	1	1	GA + 2KGA	–
*P. yamanorum* RZ5	FABCDE	1	1	GA + 2KGA	–
*Pseudomonas* sp. CCOS 191	FBCDEG	1	1	**GA** + 2KGA	Wongfun et al. (80)
*Pseudomonas* sp. GBPI_506	FABCDE	1	1	GA + 2KGA	–

^*a*^ The first 10 strains listed in the table and presented in grey constitute the collection of *Pseudomonas* strains tested *in vitro*. In the PQQ cluster composition, the letter order presents the cluster organization, and extra copies of genes are separated by a symbol “/.” In the GDH-PQQ and GADH-FAD columns, the number indicated in each cell corresponds to the predicted number of the corresponding proteins encoded in the genome of each strain (1, one copy; 0, absent). The detail of the accession numbers supporting the PQQ, GDH-PQQ, and GADH-FAD proteins is presented in Table S3. The accession numbers of the GDH-PQQ and GADH-FAD have been used to compute the maximum likelihood trees presented in Fig. 4 (GDH-PQQ) and Fig. 5 (GADH-FAD). The production of gluconate and 2-ketogluconate was predicted on the basis of the genomic organization found in each strain (presence of a single GDH-PQQ or of both a GDH-PQQ and a GADH-FAD). When experimentally demonstrated in our study and/or in previous studies, the forms of gluconate are presented in bold with the associated reference, otherwise the symbol “–” is used. In the GADH-FAD column, the symbol “–” means that no GADH-FAD was detected in the corresponding genome. The accession numbers of the genomes are listed in Table S2.

**Fig 4:**
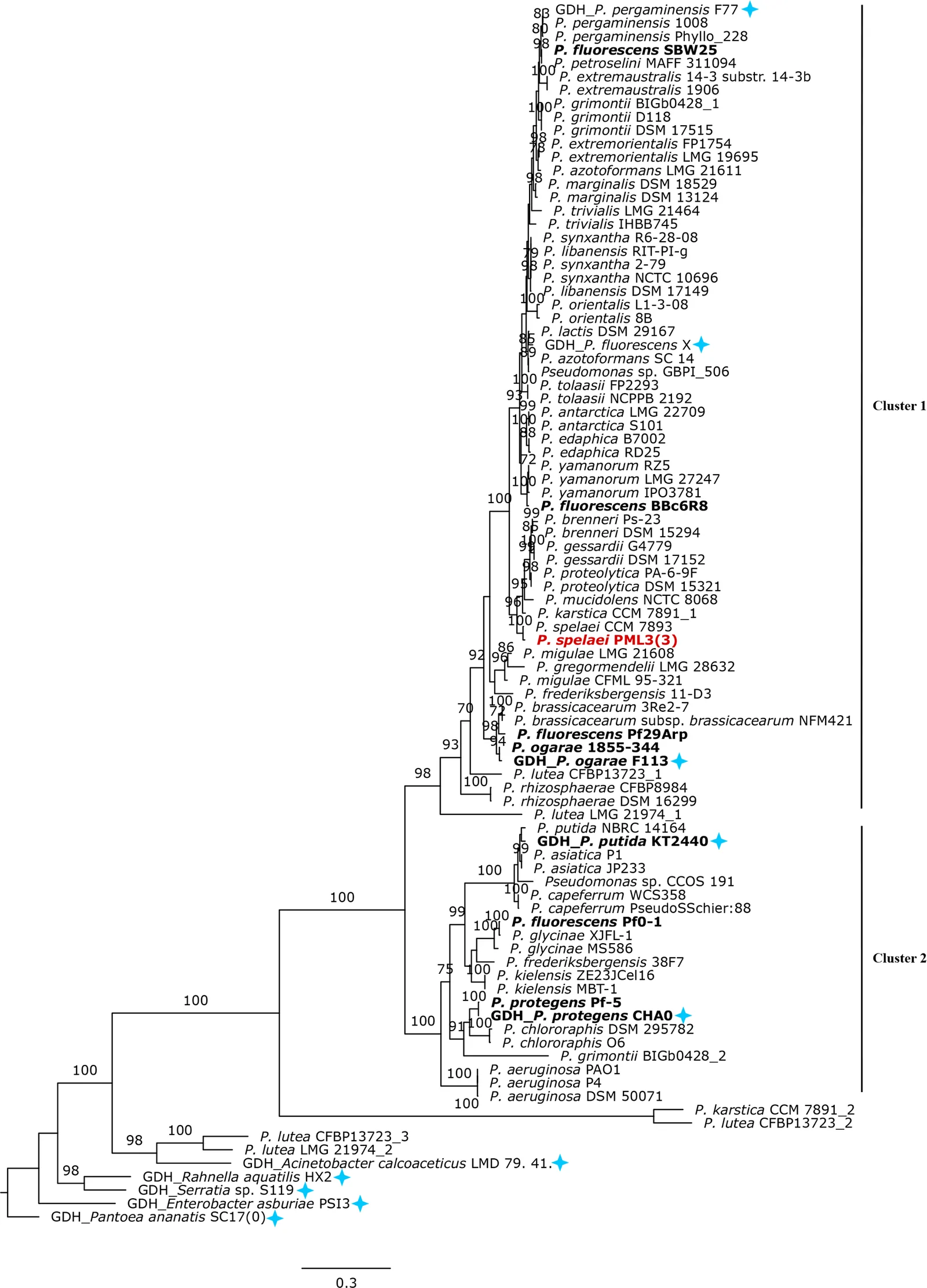
Maximum likelihood phylogenetic analysis of GDH-PQQ. A total of 91 protein sequences corresponding to demonstrated (blue cross symbol) or putative GDH-PQQ were used for the phylogenetic analysis. The GDH-PQQ of *Pantoea ananatis* SC17(0) was used as an outgroup. For the strains presenting more than one putative GDH-PQQ, this multicopy is indicated in the tree by adding after the name of the strain: (i) “_1” for the enzyme sharing the highest homology, (ii) “_2” for the second enzyme, and (iii) “_3” for the third enzyme detected. Bootstrap values under 70% were removed. The strains experimentally tested in our study are presented in bold, and the model strain PML3(3) is in red. The accession numbers of the proteins used for the construction of the tree are listed in Table 2.

**Fig 5:**
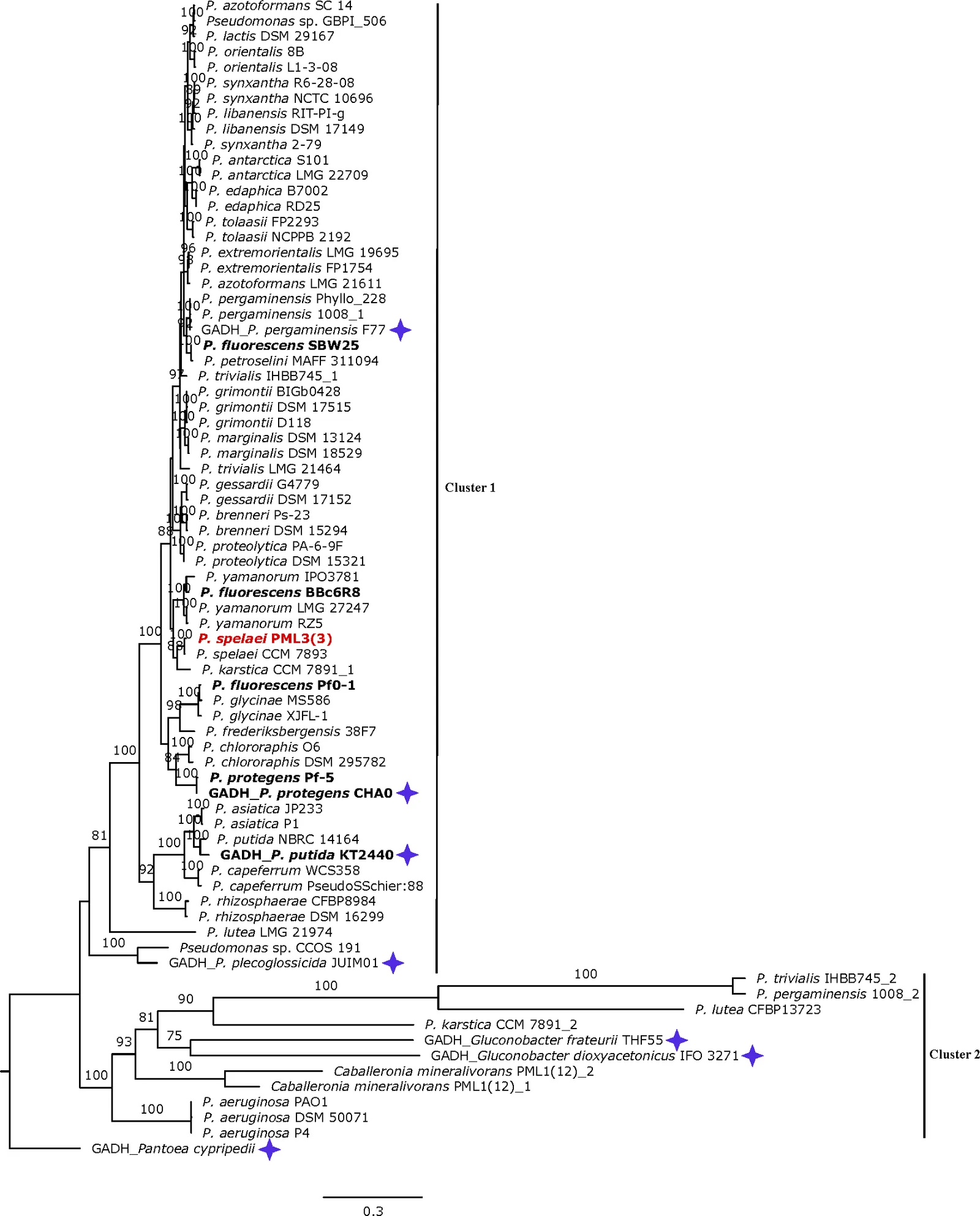
Maximum likelihood phylogenetic analysis of the GADH-FAD. A total of 75 putative GADH-FAD was used for the phylogenetic analysis. Experimentally demonstrated GADH-FAD (purple cross symbol) were also included in the tree, and the concatenated GADH-FAD protein sequence of *Pantoea cypripedii* was used as an outgroup to root the tree. When more than one putative GADH-FAD was identified in a genome, the multicopy was indicated in the tree by adding after the name of the strain: (i) “_1” for the enzyme sharing the highest homology and (ii) “_2” for the second enzyme detected after the name of the strain. Bootstrap values under 70% were removed, and two clusters comprising all the GADH-FAD were identified (i.e., Cluster 1 and Cluster 2). The 10 strains experimentally tested in our study are presented in bold, and the model strain PML3(3) is in red. The accession numbers of the proteins used for the construction of the tree are listed in Table 2.

At least one homolog of GDH-PQQ from *P. pergaminensis* F77 (AQT92806.1) was identified in all 80 genomes (Table 2). A single putative GDH-PQQ was detected in 76 genomes, sharing (65–99%) amino acid identity with the query [e.g., 92% with (MHG1903265.1) of strain PML3(3)]. Additional copies were found in four strains, with two homologs in *P. grimontii* BIGb0428, *P. karstica* CCM 7891, and *P. lutea* LMG 21974 and three homologs in *P. lutea* CFBP13723. Phylogenetic analysis grouped the putative GDH-PQQ proteins into two major clusters comprising 61 and 21 sequences (Fig. 4). The five experimentally demonstrated GDH-PQQ enzymes split between the two clusters (three in Cluster 1 and two in Cluster 2). Notably, the *Pseudomonas* GDH-PQQ homologs formed distinct clades from the experimentally characterized enzymes of other genera (e.g., *Rahnella*, *Serratia*, *Pantoea*, *Acinetobacter*, and *Enterobacter*).

A total of 66 *Pseudomonas* strains harbored homologs of the small, large, and cytochrome C subunits of the GADH-FAD of *P. plecoglossicida* JUIM01 (ATW63844.1, ATW63845.1, and ATW63846.1). This was observed for *P. spelaei* PML3(3), which shared 63%, 83%, and 73% amino acid identity with the small, large, and cytochrome C subunits *P. plecoglossicida* JUIM01, respectively. Phylogenetic analyses revealed that the putative GADH-FAD identified in the different *Pseudomonas* genomes clustered into two clusters. In Cluster 1, the sequences grouped with experimentally demonstrated GADH-FAD from *Pseudomonas* strains, whereas in Cluster 2, the sequences grouped with experimentally demonstrated GADH-FAD from other genera (*Pantoea* and *Gluconobacter*) (Fig. 5). Notably, some strains contained two copies of GADH-FAD, such as the strains *P. pergaminensis* 1008 and *P. trivialis* IHBB745.

Our analyses revealed two main genomic organizations among the 80 strains: (i) a single *gdh-PQQ* associated with a single *gadh-FAD* and (ii) a single *gdh-PQQ* alone. In addition, specific organizations were identified in six strains: (i) *P. pergaminensis* 1008 and *P. trivialis* IHBB745 harbored one *gdh-PQQ* copy and two *gadh-FAD* copies; (ii) *P. grimontii* BIGb0428 and *P. lutea* LMG 21974 harbored two *gdh-PQQ* copies and one *gadh-FAD* copy; (iii) *P. karstica* CCM 7891 harbored two *gdh-PQQ* copies and two *gadh-FAD* copies; and (iv) *P. lutea* CFBP13723 harbored three *gdh-PQQ* copies and one *gadh-FAD* copy. Among the 10 experimentally tested strains, all possessed either *gdh-PQQ* alone or both *gdh-PQQ* and *gadh-FAD*, regardless of their mineral weathering effectiveness (Table S5). The two strains unable to weather biotite (*P. ogarae* F113 and 1855-344) harbored only *gdh-PQQ*, as did the effective strain *P. fluorescens* Pf29Arp. These three strains were phylogenetically closely related on the basis of their housekeeping protein sequences and GDH-PQQ sequences (Fig. 4; Fig. S2). Notably, the most effective biotite-weathering strains, *P. fluorescens* BBc6R8 and *P. spelaei* PML3(3), were isolated from the same environment (forest) and were also closely related on the basis of organismal, GDH-PQQ, and GADH-FAD phylogenies (Fig. 4 and 5; Fig. S2; Table S6).

## DISCUSSION

Understanding the links between carbon substrate metabolism, bacterial physiology, and functional traits is essential for the development of effective biofertilizers, particularly rhizosphere bacteria that are able to solubilize phosphorus (P). This functional trait is known to rely on the production of protons and organic acids derived from carbon substrate metabolism (39, 70). However, although many bacterial strains from different genera (e.g., *Agrobacterium*, *Bacillus*, *Burkholderia*, *Collimonas*, *Caballeronia*, and *Pseudomonas*) have been considered PGPR on the basis of this ability, they have generally been tested using a single carbon source (i.e., usually glucose) and a single mineral form [i.e., usually Ca₃(PO₄)₂] (18, 45, 81). Whether the different types of carbon substrates accessible to rhizosphere bacteria in their environment (i.e., root or mycorrhizal exudates and byproducts of organic matter decomposition) influence their MWe effectiveness remains largely unknown. To address this question, we performed a comparative analysis of the MWe effectiveness, associated genetic determinants, and main organic acids produced in 10 *Pseudomonas* strains, including several model PGPR strains. By combining two types of minerals and eight to ten different carbon substrates, this study provides a comprehensive view of the relationships among carbon metabolism, bacterial physiology, and effectiveness in weathering.

The comparison of the 10 *Pseudomonas* strains grown with glucose as the sole carbon source confirmed that this substrate enabled acidification and P solubilization in all the tested strains. This result was expected, as it corresponds to the acidification-driven MWe mechanism mediated by the DO pathway of glucose described by Goldstein and Liu (37). The comparison of the solution pH and solubilization halos allowed strain ranking and revealed that the mycorrhizosphere forest strains *P. spelaei* PML3(3) and *P. fluorescens* BBc6R8 were the most effective at P solubilization (Tables S3 and S6). These observations suggest that the ecological origin of strains may influence their MWe effectiveness. Notably, high P solubilization efficiency was generally associated with limited growth, suggesting that glucose metabolism was primarily directed toward proton and gluconate production rather than biomass formation through central metabolic pathways. The detection of gluconate supported this hypothesis. Similar low biomass production during growth on liquid or solid media has been reported for several bacterial taxa and attributed to activation of the direct oxidative pathway (37). On the basis of our results and the literature, we propose a model linking the phenotypes (effectiveness in weathering vs relative growth) to the carbon substrate metabolized (Fig. S3). This model highlights how the metabolic pathway employed drives the formation of a diffuse or localized solubilization halo. Noticeably, the presence of a solubilization halo underneath the colonies has rarely been considered, as studies usually consider the ratio (halo diameter/colony diameter) >1, meaning that only solubilization halos formed around the colony were considered. In our study, considering the halos formed around and underneath the colonies reinforced the correlation between the biotite dissolution and the P solubilization data.

Screening with additional carbon substrates revealed that most mono- and disaccharides and sugar alcohols tested enabled acidification and P solubilization by the different strains. Four main phenotypic patterns were observed on the TCP plates, depending on the strain and carbon source: (i) limited growth with high P solubilization associated with strong acidification (glucose, xylose, and galactose), (ii) good growth with low P solubilization associated with moderate acidification (fructose, mannitol, sorbitol, and trehalose), (iii) strong growth without P solubilization or acidification (succinate and gluconate), and (iv) the absence of growth and activity (sucrose for most strains). These results are consistent with those of previous studies using the strains PML1(12) and F113, which reported similar carbon source-dependent phenotypes (45, 50). Similarly, *G. diazotrophicus* PAL5 showed efficient P solubilization with glucose, galactose, and xylose, but not with gluconate or fructose (82). Among the 10 *Pseudomonas* strains tested, xylose and glucose, followed by galactose, supported the highest P solubilization. Interestingly, only *P. fluorescens* SBW25 and *P. ogarae* F113, which acidified the medium with sucrose, produced visible TCP solubilization halos with this substrate. Carbon source-dependent variation in P solubilization efficiency has previously been reported for several bacterial strains, although usually with a more limited range of substrates (27, 30, 83, 84). While glucose and galactose generally supported the highest P solubilization efficiency, some strains showed higher activity with other substrates, such as xylose or maltose. For example, maltose was the most effective substrate for *Citrobacter* sp. DHRSS (maltose > glucose > sucrose > fructose) (30), whereas it did not confer P solubilization capacity to any of the ten *Pseudomonas* strains investigated here.

The biotite weathering assay provided complementary information on the MWe ability of the ten *Pseudomonas* strains. As observed for TCP solubilization, a strong relationship was found between solution pH and iron release. The same main carbon substrates (i.e., glucose, xylose, and galactose) supported the highest effectiveness in weathering biotite, indicating that the type of carbon source is a key factor controlling MWe effectiveness. Among the strains tested, the mycorrhizosphere strain *P. spelaei* PML3(3) was most effective at weathering biotite with the broadest range of carbon substrates (Table S3). Although the *Pseudomonas* strains tested in this study were less effective at weathering biotite than the reference strains *C. mineralivorans* PML1(12) and *C. pratensis* PMB3(1) in the presence of glucose, they displayed a broader range of carbon substrates supporting their weathering activity. The comparison of the two weathering assays (i.e., TCP vs biotite) revealed differences among the strains and mineral substrates. For instance, *P. ogarae* F113 and 1855-344 efficiently solubilized Ca₃(PO₄)₂ with glucose as substrate but were unable to release iron from biotite, suggesting that mineral properties and/or solution chemistry influence bacterial MWe effectiveness. Previous studies have shown that efficient acidification-driven MWe bacteria can weather various minerals, with dissolution levels depending on mineral weatherability. As an example, the strain *Collimonas pratensis* PMB3(1) has been shown to effectively weather biotite, olivine, garnet, hematite, and apatite, with a level of dissolution relying on the weatherability of the different minerals tested (83). Under our experimental conditions, Ca_3_(PO_4_)_2_ represents a highly weatherable mineral compared to biotite. This difference may be due to the physicochemical characteristics (i.e., particle size and chemical composition) of the two minerals, the solution chemistry, or the mechanism employed. We cannot exclude the possibility that the acidification developed by certain strains of *Pseudomonas* was not sufficient to allow biotite weathering and that alternative mechanisms beyond the DO pathway of glucose contribute to mineral weathering.

For most strains, differences in P solubilization effectiveness were not associated with biomass production, as the most effective strains generally showed the lowest growth. The occurrence of halos beneath or around colonies despite similar pH values suggested differences in the dynamics and localization of acidification. Notably, our kinetic analyses revealed faster acidification with glucose, with the lowest pH reached within 8 h compared with 24 h with mannitol. Similar delays in acidification have been reported for *Enterobacter asburiae* PSI3 with different substrates (84), likely reflecting differences in metabolic pathways, regulation, and enzyme localization (cytoplasm vs periplasm). Under nutrient-limited conditions, *Pseudomonas* species have been shown to bypass cytoplasmic glycolysis and redirect glucose metabolism through the periplasmic DO pathway, whereas glycolysis predominates under nutrient-rich conditions (85). Accordingly, both periplasmic and cytoplasmic glucose-metabolizing enzymes have been identified, reflecting the metabolic flexibility of these bacteria (86–88). In contrast, as with fructose, mannitol must be transported into the cytoplasm via a specific transporter and undergo metabolic interconversions before its integration into the glycolytic pathway as fructose-6-phosphate (89). In this context, the protons produced in the cytoplasm are not immediately released outside the cell, resulting in delayed acidification of the surrounding environment and lower MWe effectiveness. The absence of organic acids in the presence of mannitol compared with glucose may reflect their rapid consumption in the cytoplasm for biomass production, supporting this hypothesis.

At the molecular level, bioinformatics analyses allowed us to identify key enzymes and cofactors involved in glucose oxidation in *Pseudomonas* genomes (i.e.*,* PQQ, GDH-PQQ, and GADH-FAD) (40, 41). All 10 model strains harbored a GDH-PQQ gene, which is consistent with the results of the gluconate production observed in our study and previously reported data (45, 90). Two main gene organization patterns were identified. The first (V1) lacked *gadh-FAD* genes (e.g., *P. ogarae* F113 and 1855-344 and *P. fluorescens* Pf29Arp), whereas the second (V2) included both *gdh-PQQ* and *gadh-FAD*, enabling the conversion of glucose into gluconate and 2-ketogluconate. The production of 2KGA has already been experimentally demonstrated in some strains carrying these genes (45, 61, 76–79, 90). Regarding the strain PML3(3), our analyses revealed its ability to assimilate GA and 2KGA as sole carbon sources. Analysis of the 80 genomes revealed strong conservation of these two gene organizations, with V1 and V2 detected in 14 and 60 genomes, respectively. Six strains displayed an atypical organization, with additional copies of *gdh-PQQ* and/or *gadh-FAD*. The comparison of the genomic and experimental data revealed that GA and 2KGA production could be predicted from gene content, whereas MWe effectiveness could not be inferred solely from the presence of PQQ-, GDH-PQQ-, or GADH-FAD-encoding genes (Table 2; Table S5) (75). Notably, strains containing only *gdh-PQQ* predominantly belonged to a limited number of species, including *P. ogarae*, *P. brassicacearum*, *P. extremaustralis*, *P. kielensis*, and *P. migulae*, suggesting a phylogenetic association between strain relatedness and the absence of *gadh-FAD*. Interestingly, the strains F113, 1855-344, and Pf29Arp were able to grow in BHm containing 2KGA as the sole carbon source, indicating the presence of enzymes involved in its catabolism (data not shown). Similarly, *P. migulae* CFML 95-321 has been reported to use GA and 2KGA as the sole C source (91).

### Conclusion

This study provides new insights into the MWe ability of *Pseudomonas* and the potential of PQQ-, GDH-PQQ-, or GADH-FAD-encoding genes to predict this ability. Although the number of strains tested remains limited, the use of multiple carbon substrates and minerals highlights how the effectiveness in weathering among representatives of this genus varies, as well as the importance of the acidification dynamics according to the substrate consumed, the metabolism employed, and the type of mineral. Notably, the two mycorrhizosphere strains originating from acidic forest soils [*P. fluorescens* BBc6R8 and *P. spelaei* PML3(3)] displayed gene organizations similar to those of *Pseudomonas* strains from agricultural environments, indicating that their higher MWe effectiveness is not explained by differences in the presence or organization of the main genes involved in glucose oxidation. Instead, this difference may reflect an adaptation to oligotrophic forest soils and interactions with mycorrhizal fungi, supported by their ability to weather minerals using a broad range of carbon sources, including compounds derived from plants and fungi (i.e.*,* glucose, trehalose, and mannitol).

## Data Availability

All sequences used in this study are already available in NCBI.

## SUPPLEMENTAL MATERIAL

Supplemental figures aem.01626-26-s0001.pdf Fig. S1 to S3.

Table S1 aem.01626-26-s0002.xlsx Bacterial strains considered in the study.

Table S2 aem.01626-26-s0003.xlsx Bacterial genomes used in the study and associated data.

Tables S3 to S6 aem.01626-26-s0004.docx Strain rankings, kinetics of acidification, main organic acids, and MWe effectiveness linked to genetic basis.

ASM does not own the copyrights to Supplemental Material that may be linked to, or accessed through, an article. The authors have granted ASM a non-exclusive, world-wide license to publish the Supplemental Material files. Please contact the corresponding author directly for reuse.
