# Supplementary material for: Comparative study of the effects of different carbon substrates on acidification-driven mineral weathering by Pseudomonas
Source: Appl Environ Microbiol. 2026 Sep 22;92(10):e01626-26. doi: 10.1128/aem.01626-26 (PMC13689232; doi:10.1128/aem.01626-26)

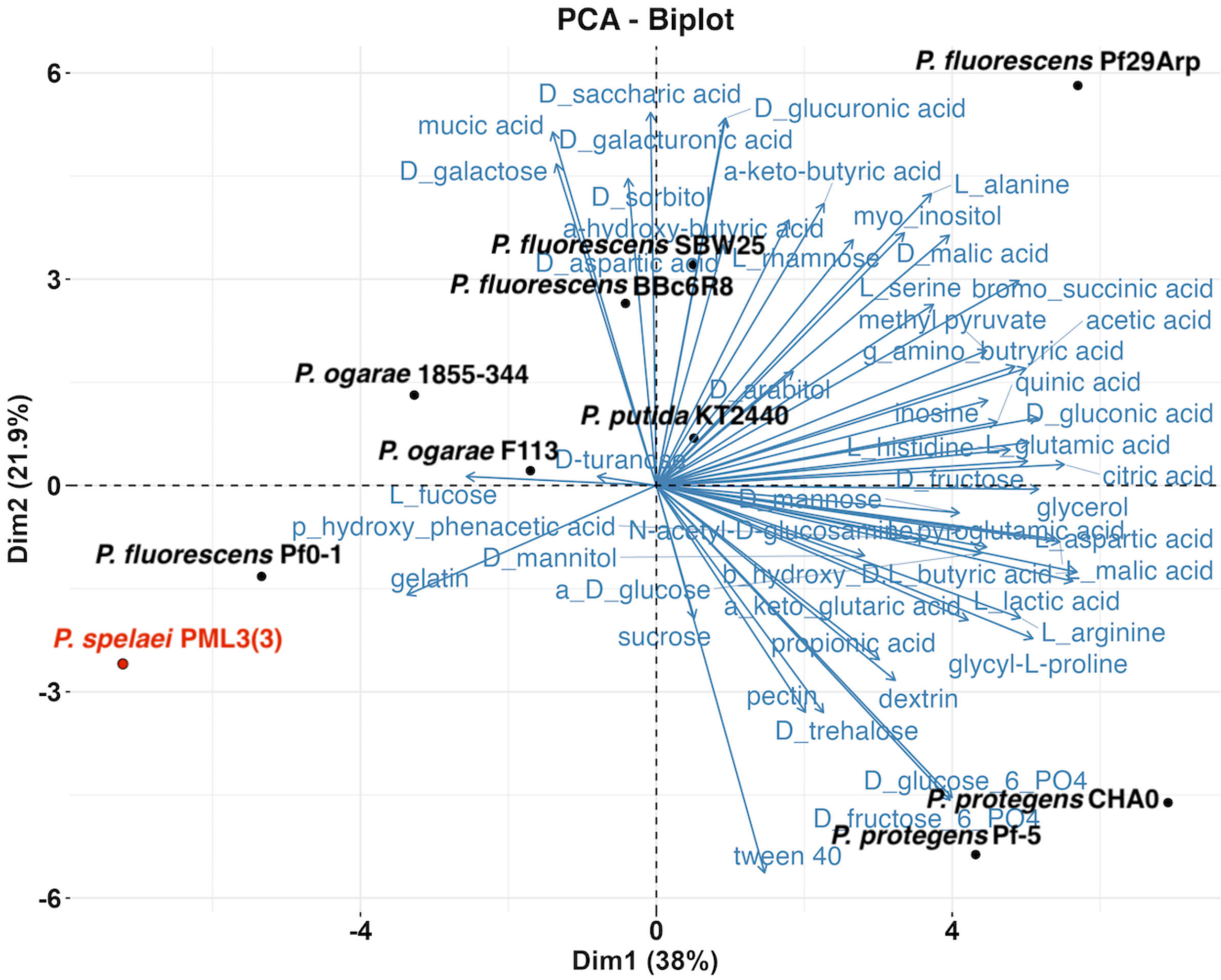

Figure S1. Metabolic fingerprint of the *Pseudomonas* collection based on the assimilation of the carbon sources tested in the GEN III microplate (Biolog), analysed through principal component analysis (PCA). The metabolic fingerprint was determined using a BIOLOG GEN Ш MicroPlates and evaluated by the measure of the O.D.$_{595nm}$ after 2 days of incubation. The carbon sources considered for this analysis were assimilated by at least one strain and are indicated in blue. The strains of the collection are presented in bold and the model strain PML3(3) in red.

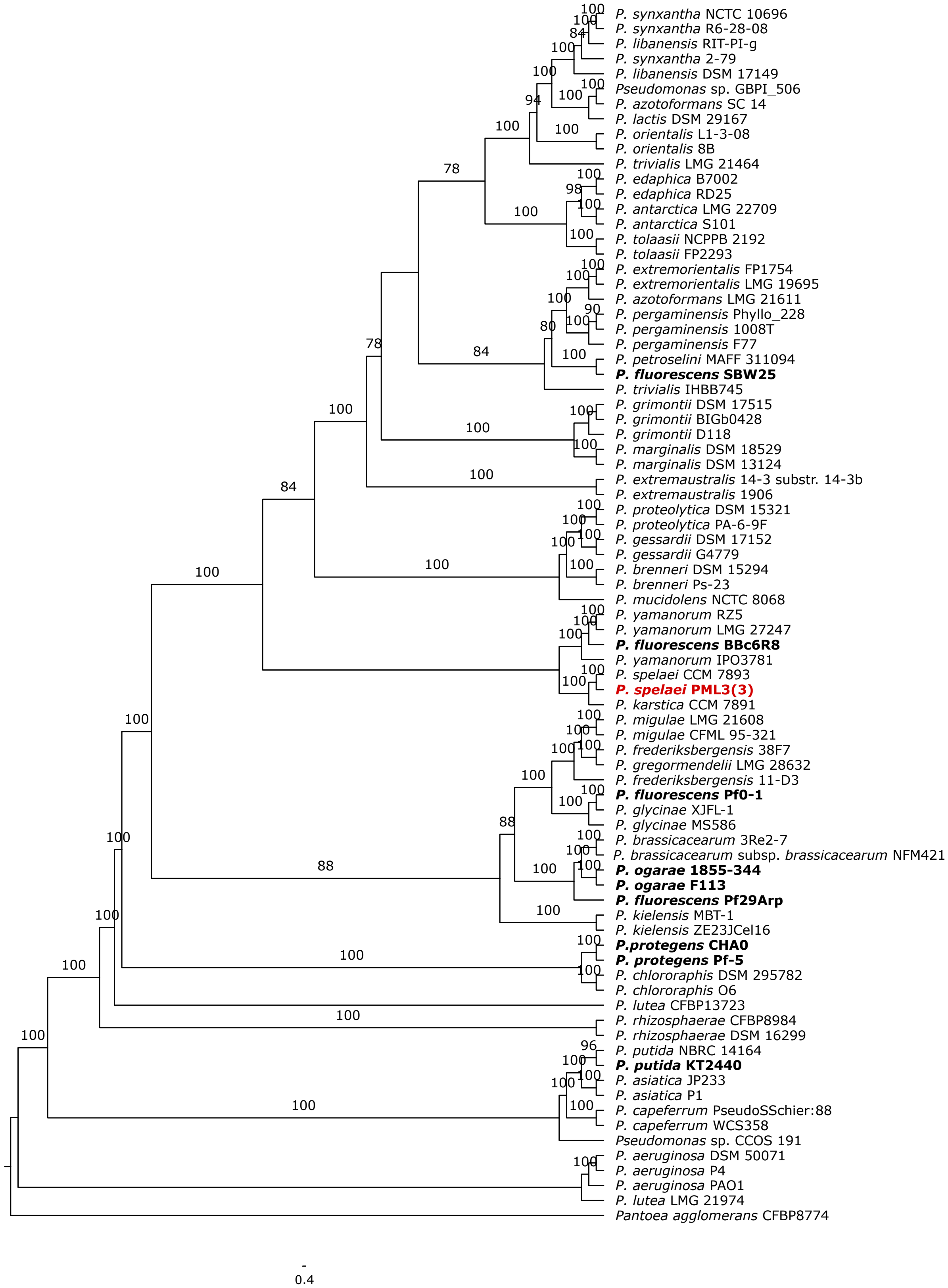

Figure S2. Organismal phylogenetic analysis of the 80 *Pseudomonas* strains. To assess the phylogenetic relationships among the 80 *Pseudomonas* strains, an organismal tree was inferred from 230 single-copy markers identified across 81 proteomes, using *Pantoea agglomerans* CFBP8774 as an outgroup. The 10 *Pseudomonas* strains from the collection are shown in bold, with the model strain PML3(3) highlighted in red bold. Bootstrap values below 70 % were removed.

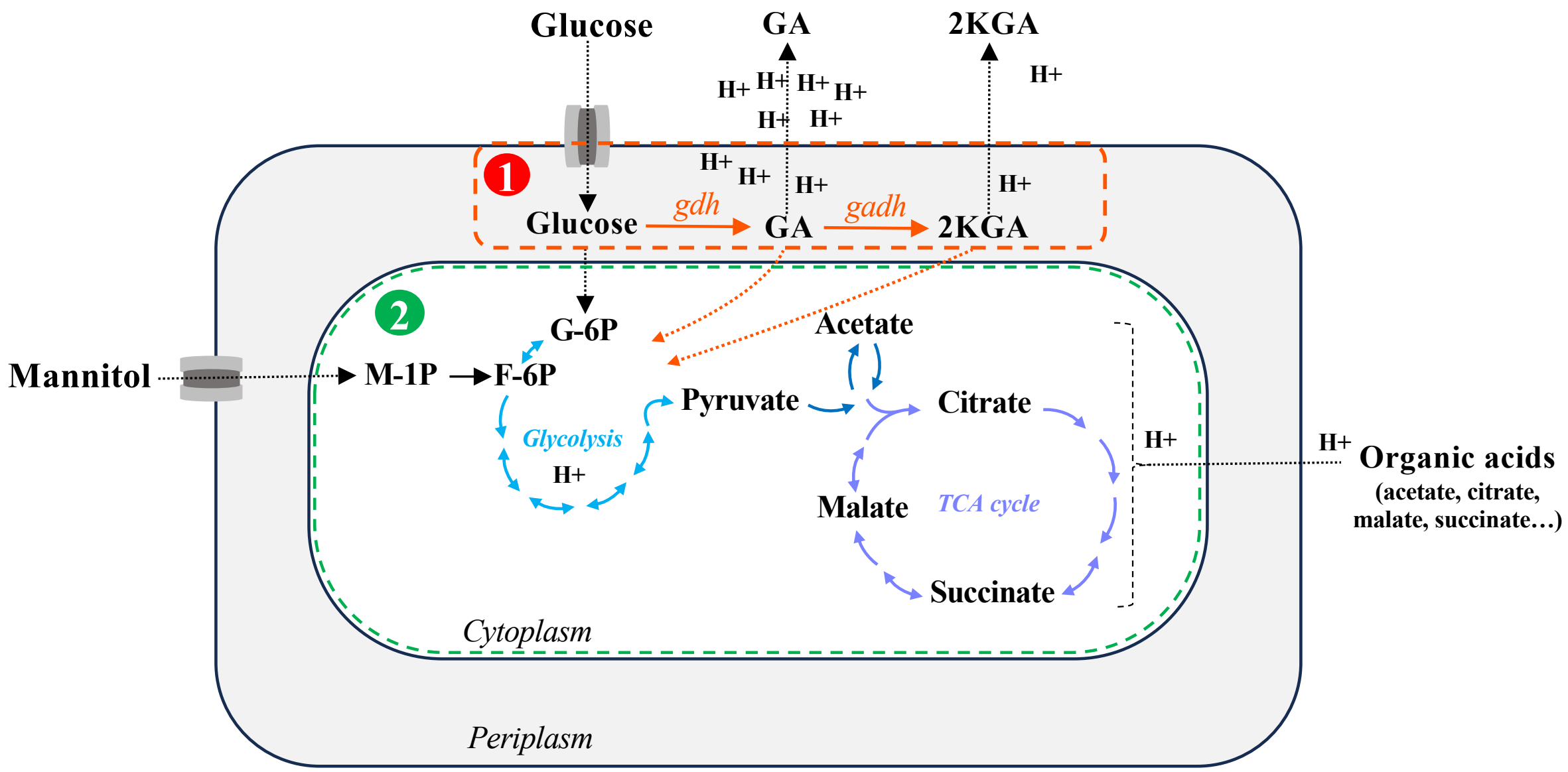

<u>**Figure S3:**</u> **Metabolic adjustments explaining the contrasting acidification-driven MWe abilities of the *Pseudomonas* strains tested.** When glucose is provided as sole carbon substrate under low nutrient availability condition, it is predominantly metabolized through the DO pathway (in red, 1) directly in the periplasm. This reaction generates organic acids (e.g., gluconate for glucose) together with proton release in the immediate vicinity of the bacterial colony, promoting a local acidification and a diffuse and efficient MWe. In contrast, the other carbon substrates (e.g., mannitol) are metabolized through central metabolism in the cytoplasm (green, 2). Mannitol is first converted to mannitol-1-phosphate and subsequently to fructose-6-phosphate before entering glycolysis (blue). Glycolytic intermediates are further connected to other metabolic pathways, including the tricarboxylic acid (TCA) cycle (purple), which also produces organic acids such as citrate. These organic acids can subsequently be exported from the cell, contributing to the acidification of the surrounding environment but to a less efficient and local MWe. These two metabolic behaviors can be observed on TCP medium and visualized by the distinct MWe patterns surrounding bacterial colonies. Red arrows present the production of acidifying molecules (protons and organic acids), while green arrows present the growth investment. The size is proportional to the effect.

1 On TCP plates in presence of glucose, xylose or galactose, the phenotype observed is characterized by a large solubilization halo around a very small colony. The low biomass production is likely explained by the metabolism of glucose through the DO pathway.

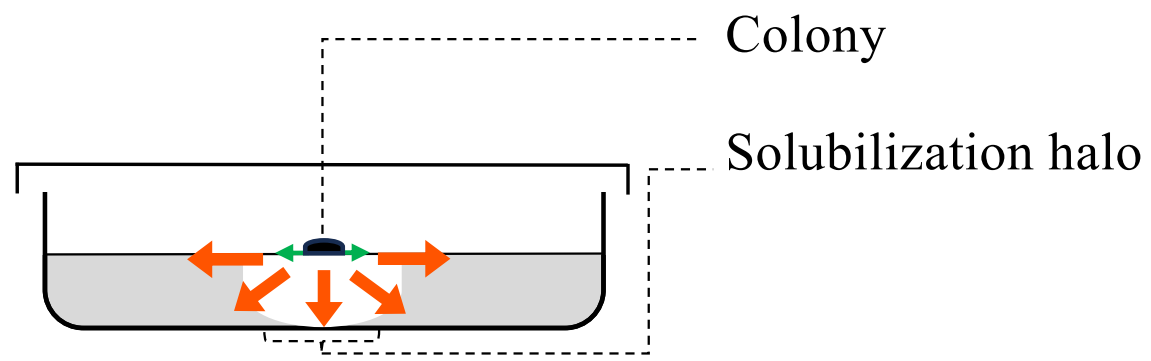

2 On TCP plates in presence of mannitol, the phenotype observed is characterized by a solubilization halo only developed underneath a large colony. The high biomass production is likely explained by the metabolism of mannitol through glycolysis, which provides large amounts of ATP and precursors for cell growth.

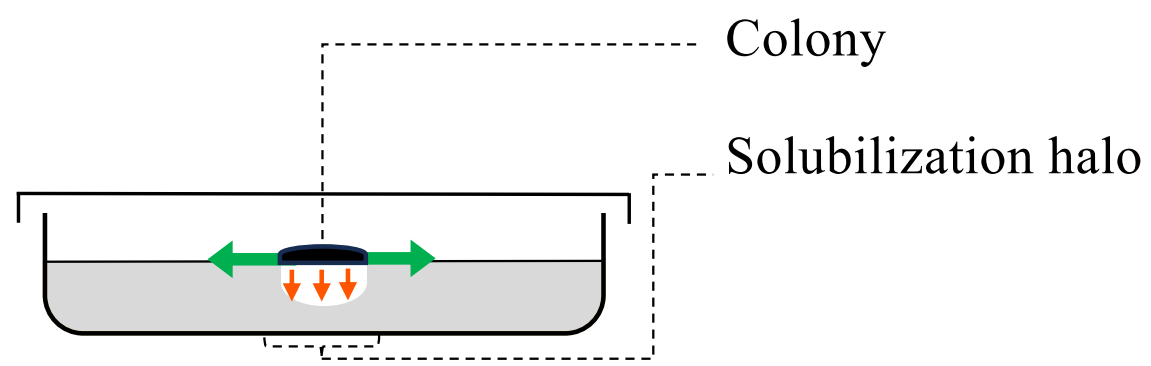

Supplement: Supplementary file 1 [file aem.01626-26-s0001.pdf]
