# Supplementary material for: Comparative study of the effects of different carbon substrates on acidification-driven mineral weathering by Pseudomonas
Source: Appl Environ Microbiol. 2026 Sep 22;92(10):e01626-26. doi: 10.1128/aem.01626-26 (PMC13689232; doi:10.1128/aem.01626-26)
Supplement: Supplementary file 2 [file aem.01626-26-s0004.docx]

**Table S3. Detail of the PQQ genes organization and accession numbers supporting the PQQ, GDH-PQQ and GADH-FAD proteins presented in Table 2.** The first ten strains listed in the table and presented in grey constitute the collection of *Pseudomonas* strains tested *in vitro*. In the PQQ cluster composition, the letter order presents the cluster organization. In the **GADH-FAD** column, the three accession numbers correspond to the small, the large, and cytochrome C subunits, respectively. Extra copies of enzymes are separated by a symbol ‘/’.

| **Strains** | ***PQQ* cluster** | **PQQ** | **GDH-PQQ** | **GADH-FAD** |
| --- | --- | --- | --- | --- |
| **Collection of 10 *Pseudomonas*** | | | | |
| ***P. spelaei* PML3(3)** | FABCDE | MHG1903827.1; MHG1903828.1; MHG1903829.1; MHG1903830.1; MHG1903831.1; MHG1903832.1 | MHG1903265.1 | MHG1898671.1; MHG1898672.1; MHG1898673.1 |
| ***P. fluorescens* BBc6R8** | FABCDE | QQD52958.1; QQD57861.1; QQD52959.1; QQD52960.1; QQD52961.1; QQD57862.1 | QQD54427.1 | QQD53530.1; QQD53531.1; QQD53532.1 |
| ***P. fluorescens* Pf29Arp** | FBCDEG | WP_030139248.1; WP_030139249.1; WP_030139250.1; WP_030139251.1; WP_030139252.1; WP_030139253.1 | WP_030138549.1 | - |
| ***P. fluorescens* Pf0-1** | FABCDEG | ABA76893.1; ABA76894.1; ABA76895.1; ABA76896.1; ABA76897.1; ABA76898.1; ABA76899.1 | ABA76314.1 | ABA71830.1; ABA71829.1; ABA71828.1 |
| ***P. fluorescens* SBW25** | FABCDE | CAY52880.1; CAY52882.1; CAY52884.1; CAY52885.1; CAY52887.1; CAY52889.1 | CAY47349.1 | CAY46336.1; CAY46337.1; CAY46338.1 |
| ***P. ogarae* F113** | FABCDEG / A | ACT32388.1; ACT32389.1; ACT32390.1; ACT32391.1; ACT32392.1; ACT32393.1; ACT32394.1 /  ACT32403.1 | ACT32412.1 | - |
| ***P. ogarae* 1855-344** | FBCDEG | KKA06130.1; KKA06129.1; KKA06128.1; KKA06127.1; KKA06126.1; KKA06125.1 | KKA05568.1 | - |
| ***P. protegens* CHA0** | FABCDEG / AB | VAV71873.1; VAV71874.1; VAV71875.1; VAV71876.1; VAV71877.1; VAV71878.1; VAV71879.1 /  AGL84055.1; VAV68562.1 | VAV71164.1 | VAV66357.1; VAV66358.1; VAV66359.1 |
| ***P. protegens* Pf-5** | FABCDEG / AB | AAY94863.1; AAY96266.1; AAY94864.1; AAY94865.1; AAY94866.1; AAY94867.1; AAY94868.1 / AAY96267; AAY91497.1 | AAY94145.1 | AAY95471.1; AAY95472.1; AAY95473.1 |
| ***P. putida* KT2440** | FABCDEG / AD | AAN66012.1; AAN66011.1; AAN66010.1; AAN66009.1; AAN66008.1; AAN66007.1; AAN66006.1 /  *pqqA*; AAN68289.1 | AAN67066.1 | AAN68988.1; AAN68987.1; AAN68986.1 |
| ***P. aeruginosa* DSM 50071** | F(distal) ABCDEG | MCP1566970.1; MCP1566983.1; MCP1566984.1; MCP1566985.1; MCP1566986.1; MCP1566987.1; MCP1566988.1 | MCP1567295.1 | MCP1567269.1; MCP1567270.1; MCP1567271.1 |
| ***P. aeruginosa* P4** | F(distal) ABCDEG | XTJ22093.1; XTJ27966.1; XTJ22080.1; XTJ22079.1; XTJ22078.1; XTJ22077.1; XTJ22076.1 | XTJ27732.1 | XTJ27757.1; XTJ27756.1; XTJ27755.1 |
| ***P. aeruginosa* PAO1** | F(distal) ABCDEG | AAG05361.1; AAG05373.1; AAG05374.1; AAG05375.1; AAG05376.1; AAG05377.1; AAG05378.1 | AAG05678.1 | AAG05652.1; AAG05653.1; AAG05654.1 |
| ***P. antarctica* LMG 22709** | FBCDE /  C | SDM86072.1, SDM86057.1; SDM86038.1; SDM86038.1; SDM85999.1 /  SDN14285.1 | SDN72665.1 | SDM72429.1; SDM72406.1; SDM72387.1 |
| ***P. antarctica* S101** | FABCDE | MFL1495300.1; MFL1495301.1; MFL1495302.1; MFL1495303.1; MFL1495304.1; MFL1495305.1 | MFL1494791.1 | MFL1493711.1; MFL1493712.1; MFL1493713.1 |
| ***P. asiatica* JP233** | FABCDEG/ AD | UYP82981.1; UYP85054.1; UYP82980.1; UYP82979.1; UYP82978.1; UYP82977.1; UYP82976.1 /  UYP85106.1; UYP85107.1 | UYP81630.1 | UYP80346.1; UYP80345.1; UYP80344.1 |
| ***P. asiatica* P1** | FABCDEG / AD | WDM89827.1; WDM91162.1; WDM89826.1; WDM89825.1; WDM89824.1; WDM89823.1; WDM89822.1 /  WDM91232.1; WDM91233.1 | WDM88373.1 | WDM86871.1; WDM86870.1; WDM86869.1 |
| ***P. azotoformans* LMG 21611** | FABCDE | SDO13884.1; SDO13913.1; SDO13947.1; SDO13981.1; SDO14015.1; SDO14050.1 | SDO80655.1 | SDO37690.1; SDO37739.1; SDO37781.1 |
| ***P. azotoformans* SC 14** | FBCDE | RXE51909.1; RXE51908.1; RXE51907.1; RXE51906.1; RXE51905.1 | RXE53670.1 | RXE52758.1; RXE52759.1; RXE52760.1 |
| ***P. brassicacearum* 3Re2-7** | FABCDEG / A | QEO81310.1; QEO81995.1; QEO81311.1; QEO81312.1; QEO81313.1; QEO81314.1; QEO81315.1 / QEO81906.1 | QEO80637.1 | - |
| ***P. brassicacearum subsp. brassicacearum* NFM421** | FBCDEG / BCDE | AEA71635.1; AEA71636.1; AEA71637.1; AEA71638.1; AEA71639.1; AEA71640.1 / AEA68285.1; AEA68286.1; AEA68287.1; AEA68288.1 | AEA70945.1 | - |
| ***P. brenneri* DSM 15294** | FBCDE | TWR75747.1; TWR75748.1; TWR75749.1; TWR75750.1; TWR75751.1 | TWR81203.1 | TWR75075.1; TWR75074.1; TWR75073.1 |
| ***P. brenneri* Ps-23** | FABCDE | MBF8003647.1; MBF8003648.1; MBF8003649.1; MBF8003650.1; MBF8003651.1; MBF8003652.1 | MBF8006293.1 | MBF8008381.1; MBF8008380.1; MBF8008379.1 |
| ***P. capeferrum* PseudoSSchier:88** | FBCDEG / DE and two copies of partial pqqC gene | WP_339470727.1; WP_033703075.1; WP_047583697.1; WP_033703078.1; WP_033703079.1; WP_339470721.1/ WP_186656103.1; WP_258713725.1; WP_047583697.1 and second partial *pqqC* | WP_186656673.1 | WP_186653494.1; WP_186653491.1; WP_410927056.1 |
| ***P. capeferrum* WCS358** | FBCDEG / DE and one copie of partial pqqC gene | KEY85022.1, KEY85023.1; KEY85024.1; KEY85025.1; KEY85026.1; KEY85027.1 / KEY88909.1; KEY88908.1; partial *pqqC* | KEY85352.1 | KEY88545.1; KEY88544.1; KEY88543.1 |
| ***P. chlororaphis* DSM 295782** | FABCDEG | WDG81782.1; WDG82367.1; WDG81781.1; WDG81780.1; WDG81779.1; WDG81778.1; WDG81777.1 | WDG76264.1 | WDG81168.1; WDG81167.1; WDG81166.1 |
| ***P. chlororaphis* O6** | FBCDEG | EIM18479.1; EIM18469.1; EIM18432.1; EIM18486.1; EIM18446.1; EIM18462.1 | EIM13529.1 | EIM18728.1; EIM18712.1; EIM18835.1 |
| ***P. edaphica* B7002** | FABCDE | NVZ59005.1; NVZ59006.1; NVZ59007.1; NVZ59008.1; NVZ59009.1; NVZ59010.1 | NVZ55608.1 | NVZ59377.1; NVZ59378.1; NVZ59379.1 |
| ***P. edaphica* RD25** | F(distal) ABCDE | TLG87475.1; TLG87869.1; TLG87865.1; TLG87866.1; TLG87867.1; TLG87868.1 | TLG91643.1 | TLG92220.1; TLG92219.1; TLG92218.1 |
| ***P. extremaustralis* 14-3 *substr.* 14-3b** | FABCDE | EZI26442.1; *pqqA*; EZI26441.1; EZI26440.1; EZI26439.1; EZI26438.1 | EZI28838.1 | - |
| ***P. extremaustralis* 1906** | FBCDE | MDF3135679.1; MDF3135680.1; MDF3135681.1; MDF3135682.1; MDF3135683.1; MDF3135684.1 | MDF3131679.1 | - |
| ***P. extremorientalis* FP1754** | FABCDE /  A | WLG56387.1; WLG59889.1; WLG56388.1; WLG56389.1; WLG56390.1; WLG56391.1/ WLG59837.1 | WLG58082.1 | WLG56978.1; WLG56979.1; WLG56980.1 |
| ***P. extremorientalis* LMG 19695** | FABCDE /  A | OIN08227.1; *pqqA*; OIN08228.1; OIN08229.1; OIN08230.1; OIN08231.1 / *pqqA* | OIN11363.1 | OIN06986.1; OIN06987.1; OIN06988.1 |
| ***[P. frederiksbergensis](https://www.ncbi.nlm.nih.gov/datasets/taxonomy/104087)* [11-D3](https://www.ncbi.nlm.nih.gov/datasets/taxonomy/104087)** | FBCDEG / BCE | PQP04955.1; PQP04954.1; PQP04953.1; PQP04952.1; PQP04951.1; PQP04950.1 / PQP00642.1; PQP00641.1; PQP00640.1 | PQP01327.1 | - |
| ***P. frederiksbergensis* 38F7** | FBCDEG / BCE | RON44505.1; RON44506.1; RON44507.1; RON44508.1; RON44509.1; RON44510.1 / RON43815.1; RON43816.1; RON43817.1 | RON43007.1 | RON55698.1; RON55697.1; RON55696.1 |
| ***P. gessardii* DSM 17152** | FABCDE | ONH45069.1; *pqqA*; ONH45070.1; ONH45071.1; ONH45072.1; ONH45073.1 | ONH44325.1 | ONH41283.1; ONH41284.1; ONH41285.1 |
| ***P. gessardii* G4779** | FABCDE | NNA96376.1; NNA96377.1; NNA96378.1; NNA96379.1; NNA96380.1; NNA96381.1 | NNA98424.1 | NNA95625.1; NNA95624.1; NNA95623.1 |
| ***P. glycinae* MS586** | FABCDEG | AMQ83459.1; AUG97647.1; AMQ83458.1; AMQ83457.1; AMQ83456.1; AMQ83455.1; AMQ87072.2 | AMQ86831.1 | AMQ82857.1; AMQ82858.1; AMQ82859.1 |
| ***P. glycinae* XJFL-1** | FABCDEG | MBX8625559.1; MBX8625558.1; MBX8625557.1; MBX8625556.1; MBX8625555.1; MBX8625554.1; MBX8625553.1 | MBX8623047.1 | MBX8624980.1; MBX8624979.1; MBX8624978.1 |
| ***P. gregormendelii* LMG 28632** | FABCDEG / ABCE | MBN3964857.1; MBN3964856.1; MBN3964855.1; MBN3964854.1; MBN3964853.1; MBN3964852.1; MBN3964851.1 /  MBN3966037.1; MBN3966036.1; MBN3966035.1; MBN3966034.1 | MBN3963839.1 | - |
| ***P. grimontii* BIGb0428** | FABCDE / BCDE | MCS3513976.1; MCS3513975.1; MCS3513974.1; MCS3513973.1; MCS3513972.1; MCS3513971.1 / MCS3514431.1; MCS3514432.1; MCS3514433.1; MCS3514434.1 | MCS3512222.1 (GDH-PQQ_1) /  MCS3511046.1 (GDH-PQQ_2) | MCS3515243.1; MCS3515244.1; MCS3515245.1 |
| ***P. grimontii* D118** | FABCDE / ABCDE | MFL1477110.1; MFL1477109.1; MFL1477108.1; MFL1477107.1; MFL1477106.1; MFL1477105.1/ MFL1482317.1; MFL1482316.1; MFL1482315.1; MFL1482314.1; MFL1482313.1 | MFL1475625.1 | MFL1477688.1; MFL1477689.1; MFL1477690.1 |
| ***P. grimontii* DSM 17515** | FABCDE | SDQ93748.1; SDQ93727.1; SDQ93700.1; SDQ93670.1; SDQ93649.1; SDQ93625.1 | SDR41704.1 | SDQ74100.1; SDQ74083.1; SDQ74059.1 |
| ***P. karstica* CCM 7891** | FABCDE | MTD19072.1; MTD19073.1; MTD19074.1; MTD19075.1; MTD19076.1; MTD19077.1 | MTD20603.1 (GDH-PQQ_1) /  MTD19647.1 (GDH-PQQ_2) | MTD17761.1; MTD17762.1; MTD17763.1 (GADH-FAD_1) /  MTD17942.1; MTD17941.1; MTD17940.1 (GADH-FAD_2) |
| ***P. kielensis* MBT-1** | FABCDEG / ABCDE | MBC2689361.1; MBC2689360.1; MBC2689359.1; MBC2689358.1; MBC2689357.1; MBC2689356.1; MBC2689355.1 /  MBC2689054.1; MBC2689055.1; MBC2689056.1; MBC2689057.1; MBC2689058.1 | MBC2690473.1 | - |
| ***P. kielensis* ZE23JCel16** | FABCDEG / ABCDE | WKL55614.1 WKL55613.1; WKL55374.1; WKL55373.1; WKL55372.1; WKL55371.1; WKL55370.1 /  WKL55706.1; WKL52566.1; WKL52565.1; WKL52564.1; WKL52563.1 | WKL50945.1 | - |
| ***P. lactis* DSM 29167** | FBCDE | KRP81708.1; KRP81645.1; KRP81644.1; KRP81643.1; KRP81642.1 | KRP77834.1 | KRP79522.1; KRP79523.1; KRP79524.1 |
| ***P. libanensis* DSM 17149** | FABCDE | KRP42738.1; *pqqA*; KRP42636.1; KRP42637.1; KRP42638.1; KRP42639.1 | KRP44696.1 | KRP47533.1; KRP47534.1; KRP47535.1 |
| ***P. libanensis* RIT-PI-g** | FBCDE | KPG77642.1; KPG77005.1; KPG77006.1; KPG77007.1; KPG77008.1 | KPG72294.1 | KPG77579.1; KPG77580.1; KPG77581.1 |
| ***P. lutea* CFBP13723** | FABCDEG | MBD8122319.1; MBD8122320.1; MBD8122321.1; MBD8122322.1; MBD8122323.1; MBD8122324.1; MBD8122325.1 | MBD8120326.1 (GDH-PQQ_1) / MBD8120504.1 (GDH-PQQ_2) / MBD8120827.1 (GDH-PQQ_3) | MBD8120654.1; MBD8120655.1; MBD8120656.1 |
| ***P. lutea* LMG 21974** | FBCDEG | SEP74888.1; SEP74857.1; SEP74824.1; SEP74790.1; SEP74755.1; SEP74725.1 | SEQ24061.1 (GDH-PQQ_1) /  SEQ65303.1 (GDH-PQQ_2) | SEP64520.1; SEP64485.1; SEP64459.1 |
| ***P. marginalis* DSM 13124** | FBCDE /  two copies of pqqA | TWR62923.1; TWR62922.1; TWR62921.1; TWR62920.1; TWR62919.1 /  TWR53716.1 / TWR63144.1 | TWR61063.1 | TWR50959.1; TWR50958.1; TWR50957.1 |
| ***P. marginalis* DSM 18529** | FABCDE /  A | TWR67512.1; TWR67571.1; TWR67511.1; TWR67510.1; TWR67509.1; TWR67508.1 / TWR69748.1 | TWR74370.1 | TWR70644.1; TWR70643.1; TWR70642.1 |
| ***P. migulae* CFML 95-321** | FBCDEG / BCDE | MCP1520882.1; MCP1520883.1; MCP1520884.1; MCP1520885.1; MCP1520886.1 /  MCP1518202.1; MCP1518203.1; MCP1518204.1; MCP1518205.1 | MCP1520329.1 | - |
| ***P. migulae* LMG 21608** | FBCDEG / BCDE | SEE97808.1; SEE97814.1; SEE97824.1; SEE97832.1; SEE97839.1; SEE97846.1 / SEE56160.1; SEE56176.1; SEE56194.1; SEE56214.1 | SEE91454.1 | - |
| ***P. mucidolens* NCTC 8068** | FABCDE /  A | SQH36810.1; SQH36813.1; SQH36814.1; SQH36816.1; SQH36818.1; SQH36820.1 / *pqqA* | SQH35692.1 | - |
| ***P. orientalis* 8B** | FBCDE | AZE86213.1; AZE86214.1; AZE86215.1; AZE86216.1; AZE86217.1 | AZE82497.1 | AZE81501.1; AZE81502.1; AZE81503.1 |
| ***P. orientalis* L1-3-08** | FBCDE | AZE91639.1; AZE91640.1; AZE91641.1; AZE91642.1; AZE91643.1 | AZE87812.1 | AZE86792.1; AZE86793.1; AZE86794.1 |
| ***P. pergaminensis* 1008** | FABCDE /  A | USW00680.1; USW04081.1; USW00681.1; USW00682.1; USW00683.1; USW00684.1 / USW03950.1 | USW02209.1 | USW01241.1; USW01242.1; USW01243.1 (GADH-FAD_1) /  USW03868.1; USW03867.1; USW03995.1 (GADH-FAD_2) |
| ***P. pergaminensis* F77** | FABCDE /  A | AQT96818.1; AQT97538.1; AQT96819.1; AQT96820.1; AQT96821.1; AQT96822.1 / AQT97424.1 | AQT92806.1 | AQT91766.1; AQT91767.1; AQT91768.1 |
| ***P. pergaminensis* Phyllo_228** | FABCDE /  A | MEX5551072.1; MEX5551071.1; MEX5551070.1; MEX5551069.1; MEX5551068.1; MEX5551067.1 / MEX5554881.1 | MEX5554043.1 | MEX5550074.1; MEX5550075.1; MEX5550076.1 |
| ***P. petroselini* MAFF 311094** | FABCDE | MCD7045445.1; MCD7045446.1; MCD7045447.1; MCD7045448.1; MCD7045449.1; MCD7045450.1 | MCD7047528.1 | MCD7043068.1; MCD7043067.1; MCD7043066.1 |
| ***P. proteolytica* DSM 15321** | FBCDE | TWR85672.1; TWR85671.1; TWR85670.1; TWR85669.1; TWR85668.1 | TWR86319.1 | TWR77104.1; TWR77105.1; TWR77106.1 |
| ***P. proteolytica* PA-6-9F** | FABCDE | MCF5059243.1; MCF5059242.1; MCF5059241.1; MCF5059240.1; MCF5059239.1; MCF5059238.1 | MCF5060375.1 | MCF5058942.1; MCF5058941.1; MCF5058940.1 |
| ***P. putida* NBRC 14164** | FBCDEG | BAN52263.1; BAN52262.1; BAN52261.1; BAN52260.1; BAN52259.1; BAN52258.1 | BAN52847.1 | BAN54626.1; BAN54625.1; BAN54624.1 |
| ***P. rhizosphaerae* CFBP8984** | FABCDEG | MEB2870924.1; MEB2870923.1; MEB2870922.1; MEB2870921.1; MEB2870920.1; MEB2870919.1; MEB2870918.1 | MEB2871537.1 | MEB2870514.1; MEB2870513.1; MEB2870512.1 |
| ***P. rhizosphaerae* DSM 16299** | FBCDEG | AIS19216.1; AIS19215.1; AIS19214.1; AIS19213.1; AIS19212.1; AIS19211.1 | AIS15892.1 | AIS18814.1; AIS18813.1; AIS18812.1 |
| ***P. spelaei* CCM 7893** | FABCDE | MUF06249.1; MUF06250.1; MUF06251.1; MUF06252.1; MUF06253.1; MUF06254.1 | MUF02756.1 | MUF03877.1; MUF03876.1; MUF03875.1 |
| ***P. synxantha* 2-79** | FABCDE | KIR21179.1; KIR21180.1; KIR21181.1, KIR21182.1; KIR21183.1; KIR21184.1 | KIR22205.1 | KIR22599.1; KIR22600.1; KIR22601.1 |
| ***P. synxantha* NCTC 10696** | FABCDE | VTR05504.1; VTR05505.1; VTR05506.1; VTR05507.1; VTR05508.1; VTR05509.1 | VTR03971.1 | VTQ86381.1; VTQ86388.1; VTQ86395.1 |
| ***P. synxantha* R6-28-08** | FABCDE | AZE69699.1; AZE69700.1; AZE69701.1; AZE69702.1; AZE69703.1; AZE69704.1 | AZE65421.1 | AZE64286.1; AZE64287.1; AZE64288.1 |
| ***P. tolaasii* FP2293** | FABCDE | WLH51515.1; WLH54853.1; WLH51516.1; WLH51517.1; WLH51518.1; WLH51519.1 | WLH53045.1 | WLH52068.1; WLH52069.1; WLH52070.1 |
| ***P. tolaasii* NCPPB 2192** | FABCDE | PKA74066.1; PKA74067.1; PKA74068.1; PKA74069.1; PKA74070.1; PKA74071.1 | PKA75716.1 | PKA74642.1; PKA74643.1; PKA74644.1 |
| ***P. trivialis* IHBB745** | FABCDE | AKS07759.1; AKS10192.1; AKS07758.1; AKS07757.1; AKS07756.1; AKS07755.1 | AKS06158.1 | AKS07209.1; AKS07208.1; AKS07207.1 (GADH-FAD_1) /  AKS05192.1; AKS05193.1; AKS05194.1 (GADH-FAD_2) |
| ***P. trivialis* LMG 21464** | FBCDE | WP_057006654.1; WP_057006653.1; WP_057006652.1; WP_057006651.1; WP_057006650.1 | WP_057010409.1 | WP_057006196.1; WP_057006197.1; WP_057006198.1 |
| ***P. yamanorum* IPO3781** | FABCDE | NWE74578.1; NWE74577.1; NWE74576.1; NWE74575.1; NWE74574.1; NWE74573.1 | NWE77534.1 | NWE73980.1; NWE73979.1; NWE73978.1 |
| ***P. yamanorum* LMG 27247** | FABCDE | SDU28707.1; SDU28696.1; SDU28687.1; SDU28675.1; SDU28666.1; SDU28655.1 | SDU10776.1 | SDU21439.1; SDU21429.1; SDU21416.1 |
| ***P. yamanorum* RZ5** | FABCDE | XUK70794.1; XUK70795.1; XUK70796.1; XUK70797.1; XUK70798.1; XUK70799.1 | XUK65799.1 | XUK64824.1; XUK64825.1; XUK64826.1 |
| ***Pseudomonas* sp. CCOS 191** | FBCDEG | CRI59481.1; CRI59482.1; CRI59483.1; CRI59484.1; CRI59485.1; CRI59486.1 | CRI58693.1 | CRI56887.1; CRI56888.1; CRI56889.1 |
| ***Pseudomonas* sp. GBPI_506** | FABCDE | MCP9730821.1; MCP9730822.1; MCP9730823.1; MCP9730824.1; MCP9730825.1; MCP9730826.1 | MCP9736370.1 | MCP9734744.1; MCP9734745.1; MCP9734746.1 |

**Table S4. Comparison of the acidification kinetics slopes when *Pseudomonas* strains are grown in BHm medium amended with glucose or mannitol.** The acidification kinetics were determined by measuring the pH of culture supernatants at different timepoints. All the slopes have been expressed as absolute values considering the first 8 hours of incubation. For each condition, the slope was estimated by fitting a linear model (*i.e*., *y = ax + b*) to the pH values obtained at 0, 4, and 8 h of incubation, with the slope (coefficient *a)* corresponding to the average rate of pH change over the incubation period.

|  | **BHm + Glucose** | **BHm + Mannitol** |
| --- | --- | --- |
| Strains and **time of incubation** | **0 - 8 h** | **0 - 8 h** |
| Abiotic | 0.0 | 0.0 |
| *P. spelaei* PML3(3) | 0.3 | 0.0 |
| *P. fluorescens* BBc6R8 | 0.3 | 0.0 |
| *P. fluorescens* Pf29Arp | 0.3 | 0.1 |
| *P. fluorescens* Pf0-1 | 0.3 | 0.0 |
| *P. fluorescens* SBW25 | 0.3 | 0.0 |
| *P. ogarae* F113 | 0.3 | 0.0 |
| *P. ogarae* 1855-344 | 0.3 | 0.1 |
| *P. protegens* CHA0 | 0.4 | 0.0 |
| *P. protegens* Pf-5 | 0.3 | 0.0 |
| *P. putida* KT2440 | 0.3 | 0.0 |
| Range | 0.3 - 0.4 | 0.0 - 0.1 |

**Table S5. Organic acids production by the collection of *Pseudomonas* strains.** The production and characterisation of the main organic acids produced was done after a 72 hours incubation in BHm medium supplemented with glucose. The main organics acids detected by the method were: formate, gluconate, butyrate, pyruvate, citrate, oxalate, propionate, acetate, succinate, malate, lactate and malonate. Only acids for which a production was detected in at least one bacterial supernatant are shown, those not detected in any supernatants were omitted. Statistical analyses were carried out and differences among triplicate sample means were analyzed using ANOVA followed by Tukey’s HSD test (*P* < 0.05). Data are presented as mean ± standard deviation, and different letters (*e.g*., a, b, c, d) indicate significant differences among means. The acidification kinetics are presented in **Fig. 3**.

|  | **Organic acids (mg.L^-1^)** | | |
| --- | --- | --- | --- |
| Strains | **Gluconate** | **Butyrate** | **Lactate** |
| Abiotic | 0.0^a^ ± 0.0 | 0.0^a^ ± 0.0 | 0.0^a^ ± 0.0 |
| *P. spelaei* PML3(3) | 7.6^ab^ ± 4.7 | 3.3^ab^ ± 1.2 | 0.0^a^ ± 0.0 |
| *P. fluorescens* BBc6R8 | 13.0^bc^ ± 2.0 | 37.3^c^ ± 2.1 | 0.0^a^ ± 0.0 |
| *P. fluorescens* Pf29Arp | 9.0^b^ ± 1.0 | 15.0^b^ ± 2.6 | 0.0^a^ ± 0.0 |
| *P. fluorescens* Pf0-1 | 6.3^ab^ ± 0.6 | 62.6^d^ ± 5.1 | 0.0^a^ ± 0.0 |
| *P. fluorescens* SBW25 | 9.3^b^ ± 1.2 | 0.0^a^ ± 0.0 | 0.0^a^ ± 0.0 |
| *P. ogarae* F113 | 44.0^f^ ± 2.0 | 0.0^a^ ± 0.0 | 55.0^b^ ± 5.6 |
| *P. ogarae* 1855-344 | 20.0^cd^ ± 3.6 | 0.0^a^ ± 0.0 | 0.0^a^ ± 0.0 |
| *P. protegens* CHA0 | 14.0^bc^ ± 2.0 | 0.0^a^ ± 0.0 | 0.0^a^ ± 0.0 |
| *P. protegens* Pf-5 | 30.6^e^ ± 5.7 | 8.3^ab^ ± 0.6 | 0.0^a^ ± 0.0 |
| *P. putida* KT2440 | 26.6^de^ ± 3.1 | 93.3^e^ ± 14.2 | 0.0^a^ ± 0.0 |

**Table S6. Global overview of the MWe potential, genome organisation and ecological origin of the 10 model *Pseudomonas* strains.** The table presents the ranking of the strains according to their ability to weather biotite using glucose, the first strains being the most effective. The iron concentration [Fe] and solution pH correspond to the data obtained in the biotite weathering assay. The halo diameters (ØH) correspond to the P solubilisation observed with TCP assay. The genomic organisation predicted refers to the presence/absence of the key enzymes (*i.e*., GDH-PQQ and GADH-FAD). Statistical analyses were carried out and differences among means were analysed using ANOVA followed by Tukey’s HSD test (*P* < 0.05). Data are presented as mean ± standard deviation, and different letters (*e.g*., a, b, c, d) indicate significant differences among means. Abiotic control data are presented in **Table 1** and this condition was assigned the letter ‘a’. The strains considered as non-effective at weathering biotite are outlined in grey. The strain PML3(3) is outlined in red

|  |  | *in vitro* MWe biotests | | | *in silico* analyses |  |
| --- | --- | --- | --- | --- | --- | --- |
| **Rank** | **Strains** | **[Fe] (mg.L^-1^)**, BHm with biotite | **pH**, BHm with biotite | **ØH (cm)**, TCP | **Genomic organisation** | **Isolation source** |
| 1 | BBc6R8 | 0.8^d^ ± 0.1 | 3.5^b^ ± 0.1 | 1.6^f^ ± 0.0 | GDH-PQQ + GADH-FAD | Forest |
| 2 | PML3(3) | 0.8^cd^ ± 0.1 | 3.6^bc^ ± 0.1 | 1.2^cd^ ± 0.1 | GDH-PQQ + GADH-FAD | Forest |
| 2 | CHA0 | 0.7^cd^ ± 0.1 | 4.0^d^ ± 0.2 | 1.0^bc^ ± 0.1 | GDH-PQQ + GADH-FAD | Agricultural |
| 3 | Pf29Arp | 0.7^bcd^ ± 0.1 | 3.7^bc^ ± 0.1 | 1.0^bc^ ± 0.1 | GDH-PQQ only | Agricultural |
| 3 | Pf-5 | 0.6^bcd^ ± 0.1 | 3.8^c^ ± 0.1 | 1.0^bc^ ± 0.1 | GDH-PQQ + GADH-FAD | Agricultural |
| 4 | SBW25 | 0.6^bc^ ± 0.1 | 3.7^ef^ ± 0.0 | 1.5^ef^ ± 0.2 | GDH-PQQ + GADH-FAD | Agricultural |
| 5 | Pf0-1 | 0.5^b^ ± 0.1 | 4.4^e^ ± 0.1 | 1.2^cd^ ± 0.2 | GDH-PQQ + GADH-FAD | Agricultural |
| 5 | KT2440 | 0.5^b^ ± 0.1 | 4.4^ef^ ± 0.2 | 0.9^b^ ± 0.0 | GDH-PQQ + GADH-FAD | Agricultural |
| 6 | F113 | 0.2^a^ ± 0.1 | 4.4^ef^ ± 0.1 | 0.9^b^ ± 0.1 | GDH-PQQ only | Agricultural |
| 6 | 1855-344 | 0.2^a^ ± 0.0 | 4.7^g^ ± 0.0 | 1.4^de^ ± 0.1 | GDH-PQQ only | Agricultural |
